# The hsa_circ_0039857/miR-338-3p/RAB32 axis promotes the malignant progression of colorectal cancer

**DOI:** 10.1186/s12876-022-02622-1

**Published:** 2022-12-20

**Authors:** Pei Xu, Siying Cheng, Xianwei Wang, Shuming Jiang, Xiaoyan He, Lina Tang, Ning Wu, Zhirong Yang

**Affiliations:** Department of Pathology, People’s Hospital of De Yang City, No. 173, Section 1 of North Taishan Road, Jingyang District, Deyang, 618000 Sichuan China

**Keywords:** Colorectal cancer, hsa_circ_0039857, miR-338-3p, RAB32, Cancer progression

## Abstract

**Background:**

Colorectal cancer (CRC) is a prevalent malignancy of the gastrointestinal. Circular RNAs (circRNAs) act as important roles in CRC malignant progression. However, the role of circ_0039857 in CRC is still unclear. Therefore, this study aimed to explore the function and mechanism of hsa_circ_0039857 in the CRC.

**Methods:**

The mRNA and protein expression were measured via RT-qPCR. RNase R assay and Actinomycin D were employed to evaluate the stability of circ_0039857. Functional experiments, such as proliferation and apoptosis, were applied to study the function of circ_0039857 in CRC cells. The underlying mechanisms of circ_0039857 were then analyzed by bioinformatics, dual-luciferase reporter gene assay, RNA pull-down and rescue experiments.

**Results:**

We revealed that circ_0039857 was significantly enhanced in CRC. Circ_0039857 was stabler than linear RNA in cells and valuable for the disease diagnosis. In addition, circ_0039857 knockdown inhibited proliferation and promoted apoptosis. Mechanistically, circ_0039857 positively regulated the expression of RAB32 via sponging miR-338-3p.

**Conclusion:**

This study demonstrated that circ_0039857 knockdown suppressed CRC malignant progression through miR-338-3p/RAB32 axis. Most importantly, this will help us to better understand the circRNA network in CRC, and may find potential biomarkers and targets for CRC clinical treatment.

**Supplementary Information:**

The online version contains supplementary material available at 10.1186/s12876-022-02622-1.

## Introduction

Colorectal cancer (CRC) is a malignant tumor of the gastrointestinal [1]. Nearly 1.6 million new cases of CRC are reported worldwide every year, and the incidence is increasing year by year [2]. It is reported that there will be more than 2.2 million newly diagnosed cases of CRC by 2030, and the public health burden will increase by approximately 60% [3]. Despite widespread improvements of CRC treatments, more than 50% of patients still die from disease progression [4]. Consequently, it is urgent to further study the molecular mechanism of CRC development in order to prolong the survival time, which is also the top priority in current CRC researches.

Circular RNAs (circRNAs) neither have a 3ʹpoly(A) tail nor a 5ʹcap, which is a particular category of non-coding RNAs with a circular structure [5]. In the past, circRNAs were seen as the production of RNA mis-splicing. Nowadays, many functional circRNAs have been discovered in mammals with the characteristics of abundance, endogenous, stability, specificity, and conservation by evolving high-throughput sequencing technologies [6]. With the deepening of research, it has been found that circRNAs are not only widely affected by normal physiological processes, but also associated with the progression of diseases, such as cardiovascular diseases, tumors, etc. [7]. Especially, abnormal circRNA expression affects tumor cell migration, growth, apoptosis and other cell processes [8]. Most importantly, circRNAs may be promising molecular targets for tumor cell therapy in future [9]. Although an increasing number of studies have investigated the function of circRNAs on tumorigenesis and development, the regulatory network of circRNAs in CRC is still incomplete [10]. RANBP10 is a ubiquitously expressed and evolutionarily conserved RAN-binding protein. It is reported that RANBP10 plays an important role in cell cycle and tumor progression [11]. Circ_0039857 is formed by back splicing of RANBP10 mRNA exon. However, there are no studies on the biological function of circ_0039857 in CRC.

MicroRNAs (miRNAs) are approximately 22nt in length, which regulates the expression of post-transcriptional genes. It ultimately leads to reduced mRNA stability and translational repression by binding to specific targets in the 3'UTR region of mRNAs [12, 13]. MiR-338-3p, located on chromosome 17q25, is downregulated in various cancers [14]. MiR-338-3p has been reported to inhibit cell growth and invasion by binding to specific targets, thereby regulating the progression of human cancers, such as lung adenocarcinoma [14], neuroblastoma [15], breast cancer [16] and ovarian cancer [17]. However, the role of miR-338-3p in CRC has not been fully elucidated.

CircRNA contains many binding sites of miRNA, which competitively bind or sponge miRNA to regulate the expression of target mRNA. [18]. Compared with other miRNA sponges, circRNAs have rich miRNA response elements (MERs) and excellent ability to bind miRNAs, which is the reason called “super sponges” [19]. Studies revealed that circRNAs regulate tumor progression through the miRNA–mRNA axis [20]. RAB32 belongs to the GTP-binding Rab protein family and is a multifunctional vesicle-associated protein [21]. RAB32 is significantly up-regulated and acts as a tumor oncogene in various human cancers. For example, miR-30 overexpression suppresses cell malignant progression by targeting RAB32 in ovarian cancer [22]. In chronic myeloid leukemia, miR-141-5p overexpression regulates cell proliferation and apoptosis through inhibiting RAB32 [23]. Inhibition of miR-30c-5p promotes tumor progression via RAB32 in hepatocellular carcinoma [24]. However, the biological function and underlying molecular mechanisms of RAB32 in CRC development remain unknown.

Therefore, studying circRNA–miRNA–mRNA mechanisms of tumor progression is a potential field for CRC treatment and prognosis. In this study, the main purpose was to explore the effects and molecular mechanism of hsa_circ_0039857 in CRC development. Our results revealed that circ_0039857 was sharply increased in CRC. Circ_0039857 knockdown inhibited cell proliferation and promoted apoptosis by targeting the miR-338-3p/RAB32 axis. These results may provide new ideas and help to find effective targets for CRC clinical treatment.

## Materials and methods

### Sample collection

All samples were selected from CRC patients in our hospital for this study. All the enrolled patients had not received preoperative treatment and were diagnosed with CRC by histopathology. This project was supported by the Research Ethics Committee of People's Hospital of De Yang City. The study was conducted in accordance with the Helsinki declaration. Informed consent form was obtained by all participants before the tissue sample collection. The patients clinicopathological characteristics were presented in Table 1.

**Table 1:** Association between the circ_0039857 expression levels in tumor tissues and clinicopathological characteristics of colorectal cancer patients

Clinicopathologic characteristics	*n*	circ_0039857		
		Low (n = 33)	High (n = 23)	*P*-value
*Age (years)*				
< 60	19	11	12	0.1586
≥ 60	37	22	11	
*Sex*				
Male	38	22	16	0.8193
Female	28	11	7	
*Tumor size*				
< 50 mm	38	26	12	0.0359*
≥ 50 mm	28	7	11	
*Lymph node metastasis*				
Yes	26	16	10	0.7117
No	30	17	13	
*Differentiation*				
Low	4	1	3	0.2979
medium	46	29	17	
High	6	3	3	

^*^P < 0.05

### Cell culture

All cell lines (NCM460, HCT116, SW620, SW480, SW837, SW48, RKO) were purchased from Shanghai Institute of Biological Sciences, Chinese Academy of Sciences. An incubator was employed to culture all cells at 37 °C and 5% CO_2_ condition. The cells were cultured in RPMI-1640 medium (Gibco, USA) with 10% FBS (Gibco, USA) and 1% penicillin–streptomycin (Gibco, USA) solution.

### RT-qPCR

TRizol kit (Invitrogen, USA) was applied to extract total RNA. The RNA purity was then measured and OD260/OD280 ratio between 1.8 and 2.0 was used for subsequent experiments. Then, PrimeScript RT reagent Kit (Takara, Japan) was conducted to reverse transcribed mRNA. A One step miRNA RT Solution (Zhendan, Shanghai, China) was used to reverse transcribed miRNA. Subsequently, SYBR Premix Ex Taq (Beyotime, China) was employed to perform RT-qPCR on a PCR machine. The relative expression of genes was performed using the 2^−ΔΔCt^ method. GAPDH was set as the internal parameters for circRNA and mRNA. U6 was set as the internal parameters for miRNA. The primer sequences were shown in Table 2.

**Table 2:** The primer sequences of each gene for RT-qPCR

Gene	Primer sequence (5′–3′)	Accession number	annealing temperature
circ_0039857	F: CATCCAGAGGGAACCTGTGT	NM_020850	50
	R: AAGTTGGCGTCCACAATCTC		
miR-338-3p	F: TGCGGTCCAGCATCAGTGATTTTGTT	NR_029897.1	45
	R: CCAGTGCAGGGTCCGAGGT		
	RT: GTCGTATCCAGTGCAGGGTCCG		
	AGGTGCACTGGATACGACGGTCGTA		
RAB32	F: CAGGTGGACCAATTCTGCAAA	NM_006834.5	48
	R: GGCAGCTTCCTCTATGTTTATGT		
Caspase-1	F: TTTCCGCAAGGTTCGATTTTCA	NM_001257119.3	45
	R: GGCATCTGCGCTCTACCATC		
Bax	F: CCCGAGAGGTCTTTTTCCGAG	NM_001291430.2	50
	R: CCAGCCCATGATGGTTCTGAT		
Bcl-2	F: GGTGGGGTCATGTGTGTGG	NM_000633.3	45
	R: CGGTTCAGGTACTCAGTCATCC		
GAPDH	F: GGAGCGAGATCCCTCCAAAAT	NM_001357943.2	45
	R: GGCTGTTGTCATACTTCTCATGG		
U6	F: CTCGCTTCGGCAGCACA	NR_004394.1	48
	R: AACGCTTCACGAATTTGCGT		
	RT: GTCGTATCCAGTGCAGGGTCCGA		
	GGTGCACTGGATACGACTGGAACG		

### RNase R assay

The stability of circ_0039857 was assessed by RNase R treatment. RNA (10 μg) isolated from SW480 cells and RKO cells was incubated with 40 U RNase R (Epicenter, USA) for 15 min. Circular and linear RNAs expression was then analyzed by RT-qPCR.

### Actinomycin D assay

2 mM actinomycin D (Sigma, USA) was applied to treat SW480 and RKO cells for 0, 4, 8, 12, and 24 h. Next, the cells were collected. Then, at different time points, the expression of circular and linear RNAs was examined by RT-qPCR. Finally, the mRNA expression curve was plotted.

### Cell transfection

Small interfering RNAs targeting circ_0039857 (si-circ_0039857#1 and circ_0039857#2), RAB32 overexpression plasmids (OE-RAB32), miR-338-3p mimics and inhibitors, and corresponding negative controls were synthesized by Anhui General Biosynthesis. Cells were transfected by lipofectamine 3000 (Invitrogen, USA). After transfection for 48 h, the efficiency of cell transfection was evaluated via RT-qPCR. The sequences of siRNAs were as follows:

- si-circ_0039857#1: 5ʹ-AACAGCGCCATTTTAGGTATA-3’;
- si-circ_0039857#2: 5ʹ-ATTTTAGGTATAGCCTTCACA-3’;
- si-NC: 5ʹ-AGTACTTGACCTGCTTAGGCTGCA-3’.

### CCK-8 assay

The cells were collected and prepared into cell suspensions. Then, 3000 cells were added into each well of 96-well plate. After culturing in an incubator at 37 °C for designated time point (0, 24, 48, 72 h), the cell culture plate was taken out from the incubator and added 10 μl of CCK-8 working solution (Dojindo, Japan) each well. After another 1 h of cell culture, the optical density value was calculated at 450 nm.

### Clone formation experiments

Transfected SW480 and RKO cells (300 cells/well) were added to 12-well plates. Then, SW480 and RKO cells were cultured for two weeks. Fresh medium was changed every 2 days during cell culture. After macroscopic cell clones appeared, PBS was employed to wash cells twice. Next, 4% paraformaldehyde solution (Beyotime, China) was utilized to fix cells for 20 min. Subsequently, crystal violet solution (Beyotime, China) was added to stain cells for 30 min. Finally, a camera (canon EOS 600d, Japan) was applied to photograph and count the number of cell clusters.

### Apoptosis

The collected cells were resuspended in 1 × binding buffer solution and adjusted to 8 × 10^4^ cells/ml density. Then, 100 μl binding solution was incubated with 5 μl Annexin V-PE and 5 μl 7-AAD (Solarbio, China) for 15 min in the dark. Finally, flow cytometry (BD Biosciences, USA) was performed to analyze cell samples within 1 h.

### Western blot

After collecting the treated cells of each group and washing them three times using pre-cooled PBS, RIPA cell lysis buffer (Beyotime, China) was added to lyse the cells on ice. Then the supernatant solution containing total protein after centrifugation was collected. BCA kit (Beyotime, China) was utilized to determine the extracted total protein concentration. Next, proteins were separated by 10% SDS-PAGE electrophoresis in constant pressure mode. Subsequently, the target protein was transferred to PVDF membrane (Millipore, USA) and blocked with 5% skim milk for 1 h. Next, the membranes were cut according to molecular weight before hybridization with the antibody. After adding the primary antibody (abcam, USA), membrane was incubated at 4 °C overnight. Then, TBST solution was applied to wash the membrane three times and incubated with diluted HRP-conjugated secondary antibody (abcam, USA) for 1 h. Finally, the ECL chemiluminescence substrate reagent (Thermo Fisher Scientific, USA) was added, and a chemiluminescence image analyzer was employed to take pictures. As the internal reference, GAPDH was employed to normalize protein levels.

### Bioinformatics analysis

Interaction of circRNA–miRNA was predicted by the StarBase (https://starbase.sysu.edu.cn/) and circinteractome (https://circinteractome.nia.nih.gov/) online databases. miR-338-3p was a binding target of circ_0039857. Target regions of miRNA–mRNA were predicted by StarBase (https://starbase.sysu.edu.cn/) and miRDB (https://mirdb.org/) bioinformatics tools. RAB32 was a predicted target of miR-338-3p. Subsequently, the targeted binding relationships between genes were verified using dual-luciferase reporter gene assay and RNA pull-down assay.

### Dual-luciferase reporter gene assay

The dual luciferase reporter pmiR-GLO (Promega, USA) was conducted to perform luciferase assays. Circ_0039857 and RAB32 wild-type (wt) or mutant (mut) type sequences were inserted into the pmiR-GLO plasmid. 2 × 10^4^ SW480 or RKO cells were cultured in 24-well plates overnight. Lipofectamine 3000 was employed to co-transfected cells with wt or mut plasmid and miR-338-3p mimic (10 nM) or mimic control (10 nM). A dual-luciferase assay kit (Promega, USA) was performed to examine luciferase activity after 48 h of transfection.

### RNA-RNA pull down

Anhui General Biological Company was employed to synthesize biotin-labeled miR-338-3p and nc probes. Then, to obtain probe-coated beads, streptavidin magnetic beads (Life Technologies, USA) were incubated with probes for 2 h at 25 °C. Next, lysis buffer (Thermo Fisher Scientific, USA) was applied to lyse SW480 and RKO cells (1 × 10^7^). The probe-coated beads were then incubated with cell lysates overnight at 4 °C. Subsequently, RNA complexes were eluted from the beads and purified by TRIzol reagent (Takara, Japan). Finally, qRT-PCR was conducted to analyze the abundance of circ_0039857 and RAB32 enriched by biotinylated miR-338-3p.

### Statistical analysis

GraphPad software was adopted to perform statistical analysis. Student's t-test was applied to analyze differences between the two groups. In addition, a one-way analysis of variance was used to compare differences among multiple groups. Mean ± standard deviation was employed to exhibit the experiment data. *P* < 0.05 was considered as statistically significant difference.

## Results

### circ_0039857 is significantly upregulated in CRC

**Fig. 1:**
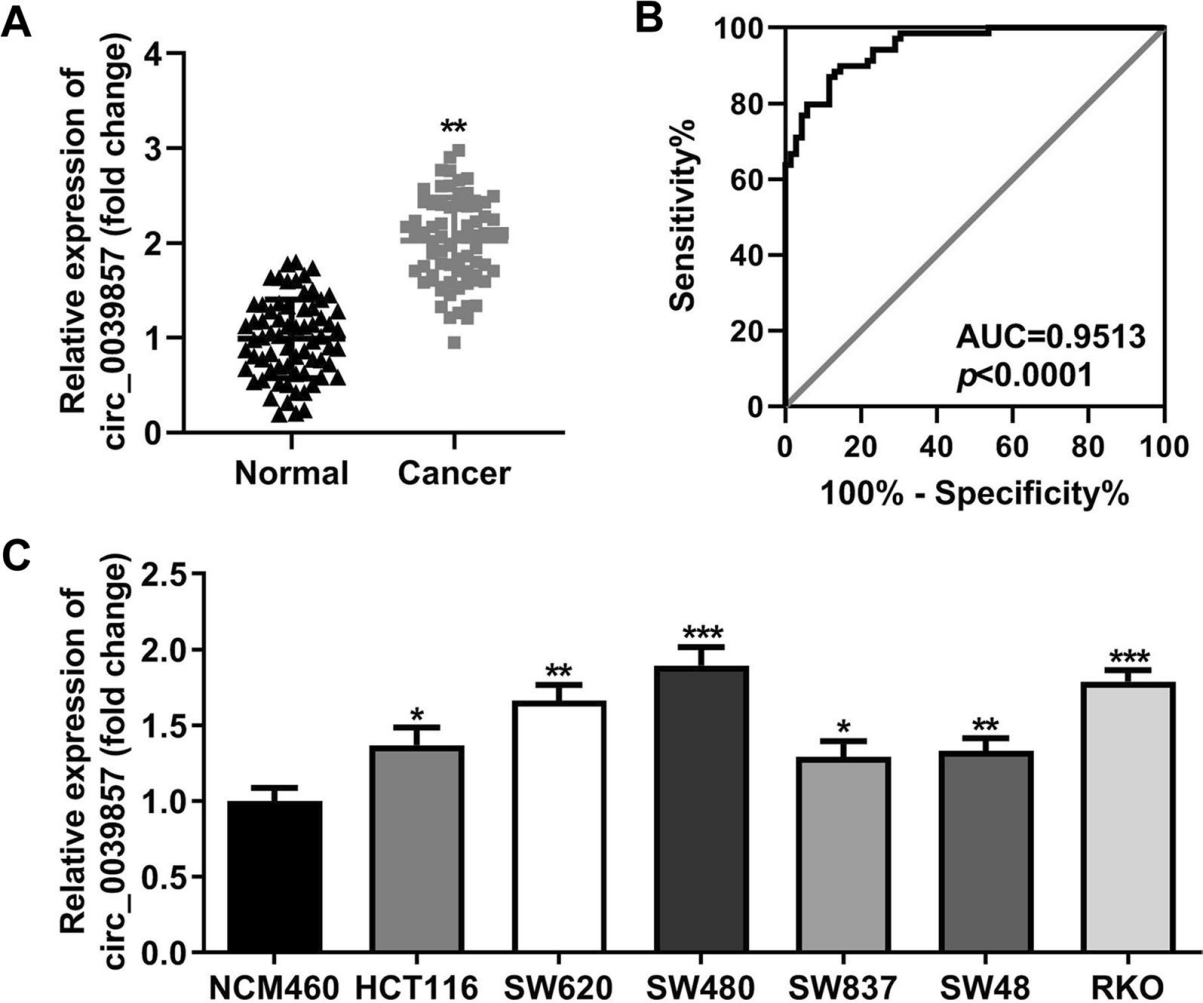
circ_0039857 is highly increased in CRC. **A** the expression level of circ_0039857 was analyzed in tissues by RT-qPCR. **B** ROC curve was applied to evaluated the diagnostic significance of circ_0039857. **C** the expression level of circ_0039857 was measured in cells by RT-qPCR. AUC, area under curve; ROC, receiver operator characteristic curve. *P < 0.05, **P < 0.01, ***P < 0.001

For the purpose of exploring potential targets of CRC treatment, RT-qPCR was employed to analyze abnormal expression of circRNA in CRC. The data revealed that circ_0039857 was obviously increased in CRC tissues (Fig. 1A). Subsequently, the diagnostic significance of this molecule for CRC was analyzed by ROC curve. The area under the curve is close to 1, indicating a higher diagnostic value. The data revealed that the AUC was 0.9513, which indicated that circ_0039857 was meaningful for the diagnosis of CRC (Fig. 1B). Next, we selected normal human colon cell line (NCM460) and CRC cell lines (HCT116, SW620, SW480, SW837, SW48 and RKO) to evaluate the expression of circ_0039857. Expectedly, circ_0039857 was obviously enhanced in CRC cell lines (Fig. 1C). Finally, SW480 and RKO cells were chosen for subsequent cell experiments due to their higher expression than other CRC cell lines.

### circ_0039857 is highly stable in CRC cells

**Fig. 2:**
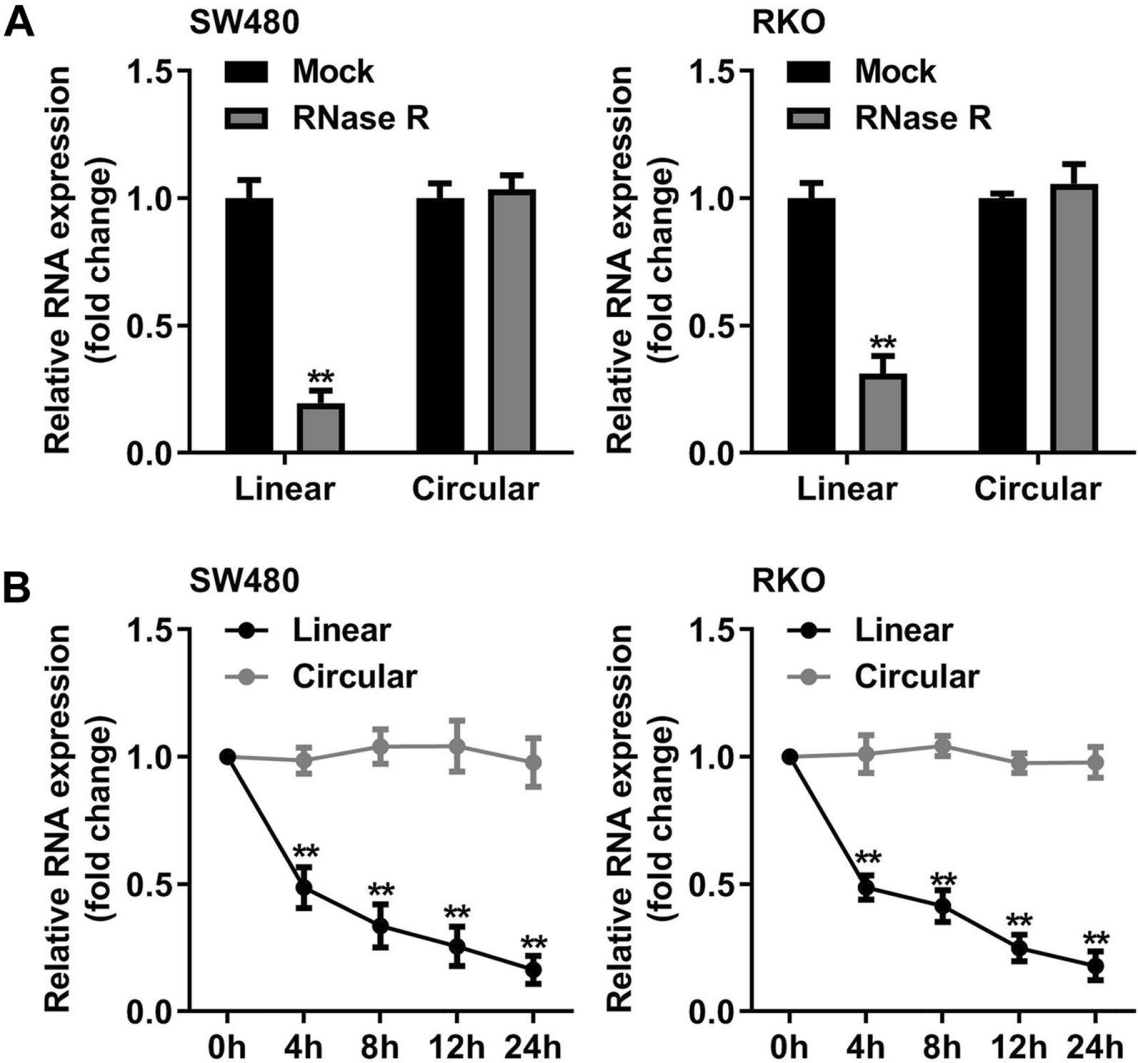
circ_0039857 is highly stable in CRC cells. **A** RNase R assay. **B** Actinomycin D assay. **P < 0.01

The stability of circ_0039857 was then verified in SW480 cells and RKO cells. RNase R results showed that circ_0039857 was against to RNase R, while RNase R was easy to digest the liner RNA (Fig. 2A). Meanwhile, actinomycin D results indicated that circ_0039857 had a longer half-life than linear RNA (Fig. 2B). These data revealed that circ_0039857 was highly stable in cells.

### Knockdown of circ_0039857 inhibits the CRC cell proliferation

**Fig. 3:**
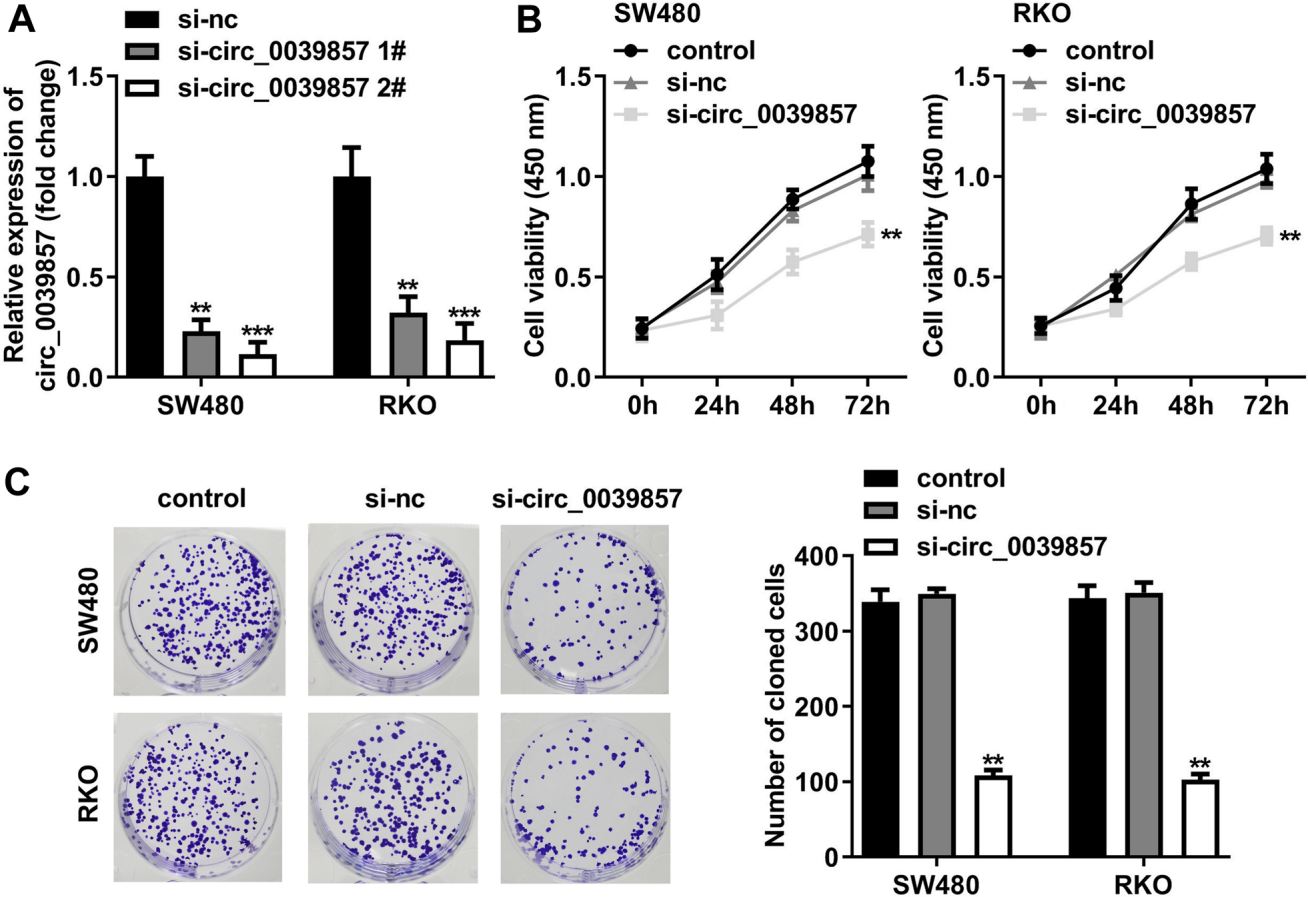
Knockdown of circ_0039857 inhibits the proliferation of CRC cells. **A** The knockdown efficiencies of si-circ_0039857#1 and circ_0039857#2 in SW480 and RKO cells were detected by RT-qPCR. **B** CCK-8 assay was performed to measure the proliferation ability of the cells. **C** The clonogenic ability of the cells was tested by plate cloning experiments. **P < 0.01, ***P < 0.001

Next, we carried out loss-of-function experiments to investigate the biological behavior of circ_0039857 in CRC cells. First, RT-qPCR was employed to detect the knockdown efficiencies of si-circ_0039857#1 and circ_0039857#2 in SW480 and RKO cells. The results revealed that circ_0039857 was more significantly decreased in cells transfected with si-circ_0039857#2 (Fig. 3A). Then, the proliferation ability of the cells was measured via the CCK-8 assay. After knockdown of circ_0039857, cell proliferation ability was uncommonly reduced (Fig. 3B). Finally, the clonogenic ability of cells was evaluated by plate cloning experiments. We observed that circ_0039857 knockdown obviously suppressed clonogenic ability (Fig. 3C). Based on these data, circ_0039857 knockdown reduced the proliferative capacity of CRC cells.

### Knockdown of circ_0039857 promotes CRC cell apoptosis of CRC

**Fig. 4:**
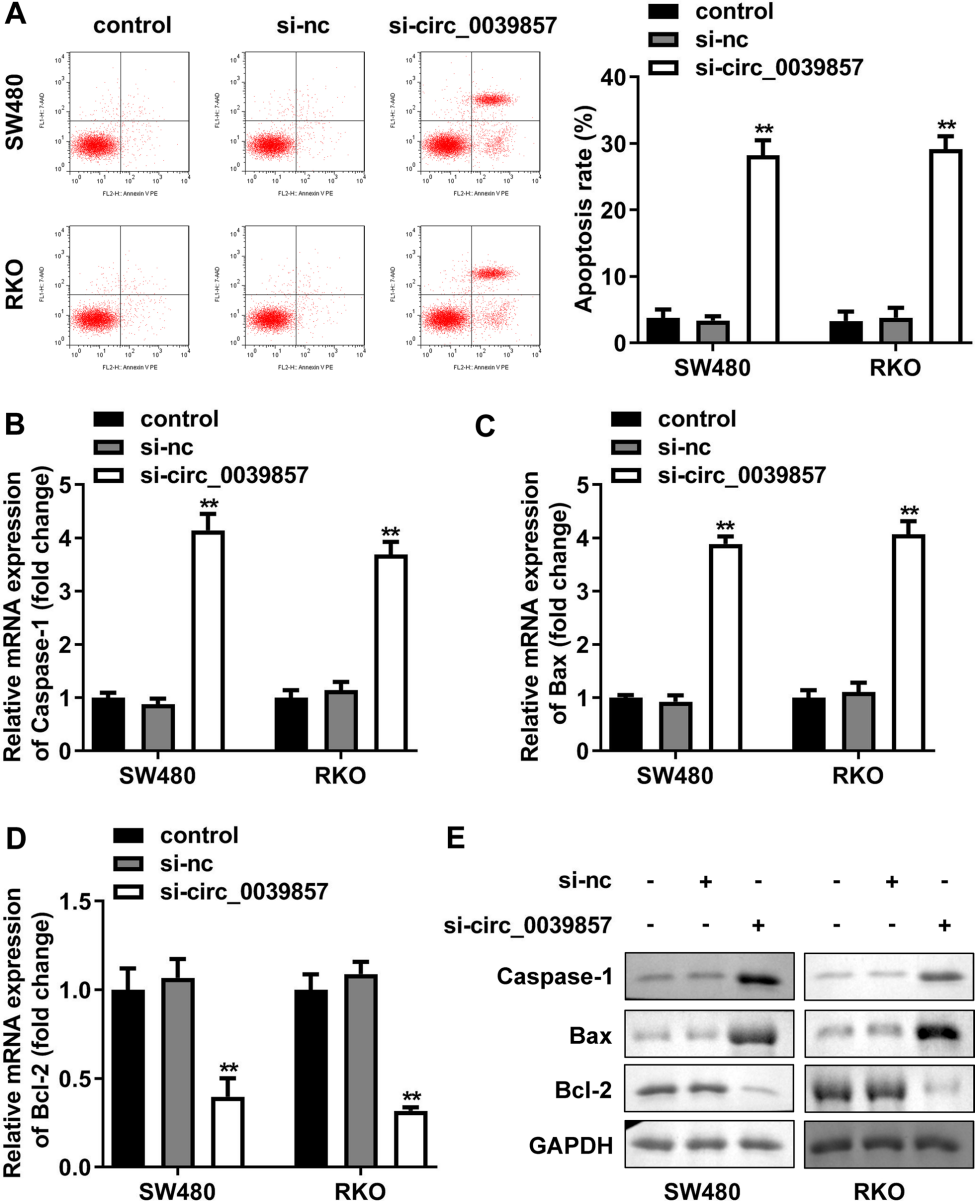
Knockdown of circ_0039857 promotes apoptosis of CRC cells. **A** The apoptosis rate of the cells was analyzed by flow cytometry. **B** The mRNA expression of caspase-1. **C** The mRNA expression of Bax. **D** The mRNA expression of Bcl-2. **E** The protein expression of caspase-1, Bax and Bcl-2. The membrane was cropped according to the molecular weight of the protein before the incubation with primary antibodies. GAPDH was used as a control. Full-length blots/gels are presented in Additional file 1: Fig. S1. **P < 0.01

The apoptosis ability of the cells was also analyzed to verify the biological function of circ_0039857. The apoptosis rate of SW480 and RKO cells was obviously increased by knockdown of circ_0039857 (Fig. 4A). Apart from that, RT-qPCR and western blot were also adopted to evaluate expression of apoptosis-related genes in cells. We observed that in circ_0039857 knockdown SW480 and RKO cells, the mRNA and protein levels of pro-apoptotic genes (caspase-1 and Bax) were clearly upregulated, while the mRNA and protein levels of anti-apoptotic gene (bcl-2) were distinctly downregulated (Fig. 4B–E). These results demonstrated that circ_0039857 knockdown strengthened the apoptotic capacity of cells.

### circ_0039857 negatively regulates miR-338-3p

**Fig. 5:**
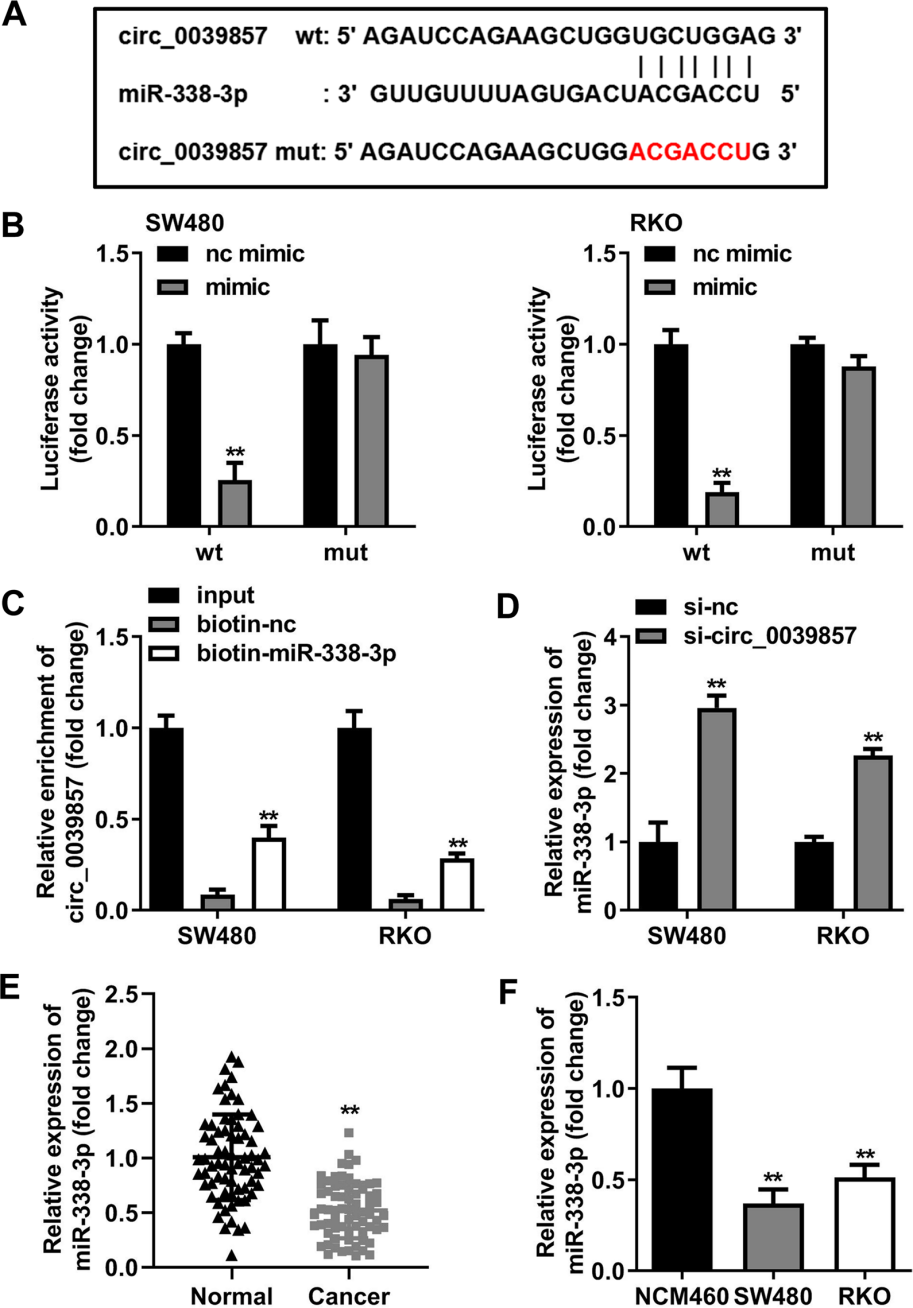
circ_0039857 negatively regulates miR-338-3p. **A** Binding sites between circ_0039857 and miR-338-3p. **B** Dual-luciferase reporter gene assay. **C** RNA pull-down. **D** The regulatory effect of circ_0039857 on miR-338-3p. **E** The expression level of miR-338-3p in tissues. **F** The expression level of miR-338-3p in cells. **P < 0.01

The potential circRNA–miRNAs regulatory mechanism of circ_0039857 was analyzed. Surprisingly, bioinformatics analysis revealed that circ_0039857 bound to miR-338-3p (Fig. 5A). Next, to verify this binding relationship, we constructed circ_0039857-wt and circ_0039857-mut vectors. These two vectors and miR-338-3p or nc mimics were then co-transfected into SW480 and RKO cells, respectively. We observed that the miR-338-3p mimic only remarkably suppressed the luciferase activity of circ_0039857-wt group cells (Fig. 5B). Subsequently, we performed RNA pull-down assay. We found that circ_0039857 was enriched in the biotin-labeled miR-338-3p group (Fig. 5C). These results fully indicated that circ_0039857 targeted and bound to miR-338-3p. Then, we verified whether circ_0039857 regulated miR-338-3p. As a result, knockdown of circ_0039857 clearly strengthened miR-338-3p expression in SW480 and RKO cells (Fig. 5D). Finally, miR-338-3p expression was also verified in CRC. Undoubtedly, miR-338-3p was notably reduced in CRC tissues (Fig. 5E) and cells (Fig. 5F). Overall, these results illustrated that circ_0039857 was negatively regulated miR-338-3p.

### circ_0039857 regulates the proliferation and apoptosis by miR-338-3p

**Fig. 6:**
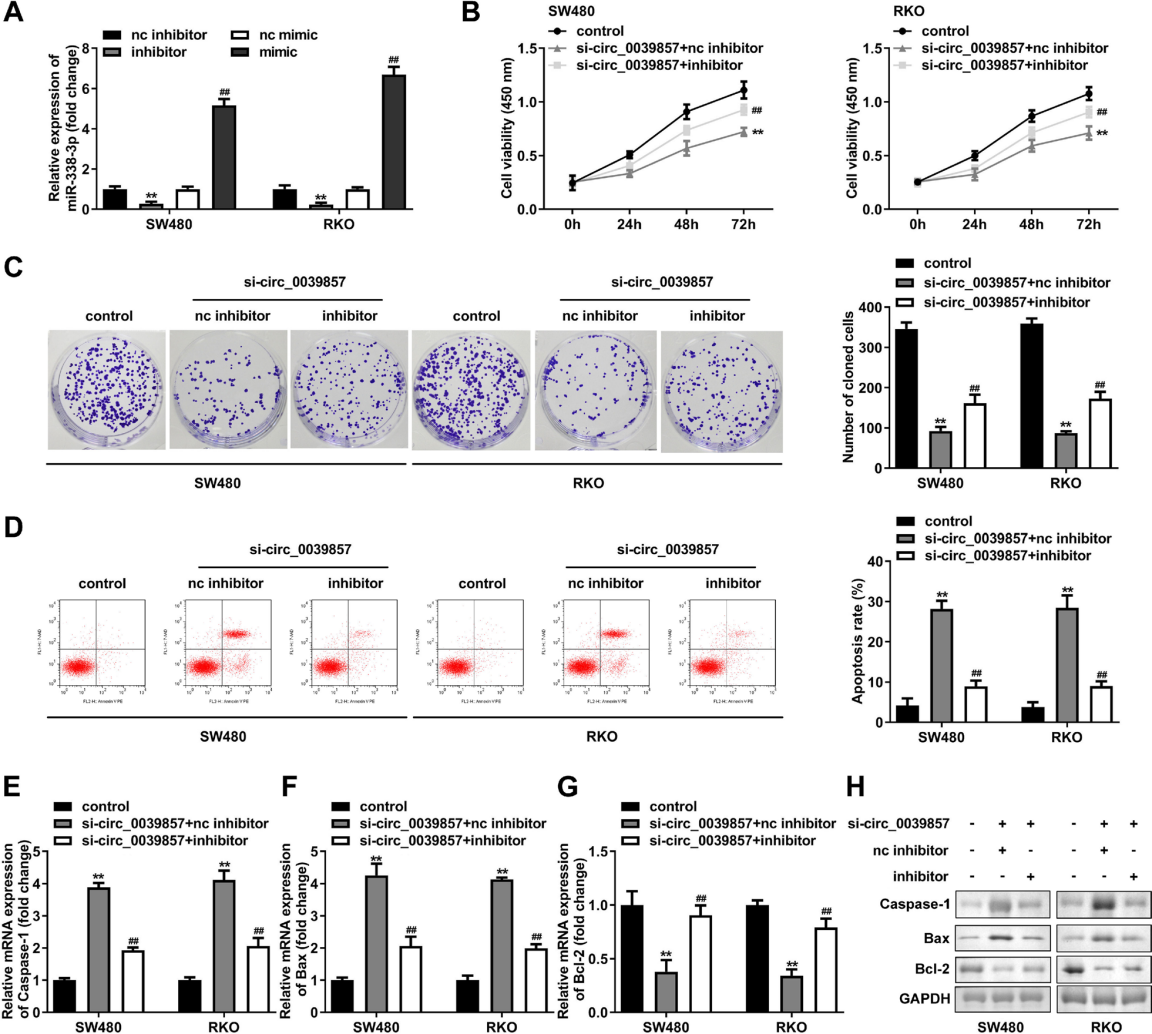
circ_0039857 regulates the proliferation and apoptosis of CRC cells via miR-338-3p. **A** The transfection efficiencies of miR-338-3p mimics or inhibitors in SW480 and RKO. **B** CCK-8 assay was performed to measure the proliferation ability of the cells. **C** The clonogenic ability of the cells was tested. **D** The apoptosis rate of the cells was analyzed by flow cytometry. **E** The mRNA expression of caspase-1. **F** The mRNA expression of Bax. **G** The mRNA expression of Bcl-2. **H** The protein expression of caspase-1, Bax and Bcl-2. The membrane was cropped according to the molecular weight of the protein before the incubation with primary antibodies. GAPDH was used as a control. Full-length blots/gels are presented in Additional file 2: Fig. S2. **P < 0.01. ##*P* < 0.01

To determine whether circ_0039857 exerts biological functions through miR-338-3p in CRC, miR-338-3p mimics or inhibitors were employed to interfere with its expression. Cells transfected with miR-338-3p inhibitors showed a reduction expression of miR-338-3p. In contrast, cells transfected with miR-338-3p mimics showed an induction miR-338-3p expression (Fig. 6A). Subsequently, si-circ_0039857 and miR-338-3p inhibitor were co-transfected into SW480 and RKO cells for rescue experiments, and NC inhibitor was used as a control. CCK-8 and clone formation experiments demonstrated that circ_0039857 knockdown obviously reduced the cell proliferation. However, simultaneous inhibition of miR-338-3p reversed the proliferation suppression of circ_0039857 knockdown (Fig. 6B and C). In addition, similar reversal effects were found in assays for apoptotic capacity. Briefly, circ_0039857 knockdown increased cell apoptosis. In contrast, when miR-338-3p was contemporarily inhibited, cell apoptosis was re-restrained (Fig. 6D–H). Taken together, these results manifested that miR-338-3p inhibition resisted the function of circ_0039857 knockdown on CRC cell behavior.

### miR-338-3p targets and negatively regulates RAB32

**Fig. 7:**
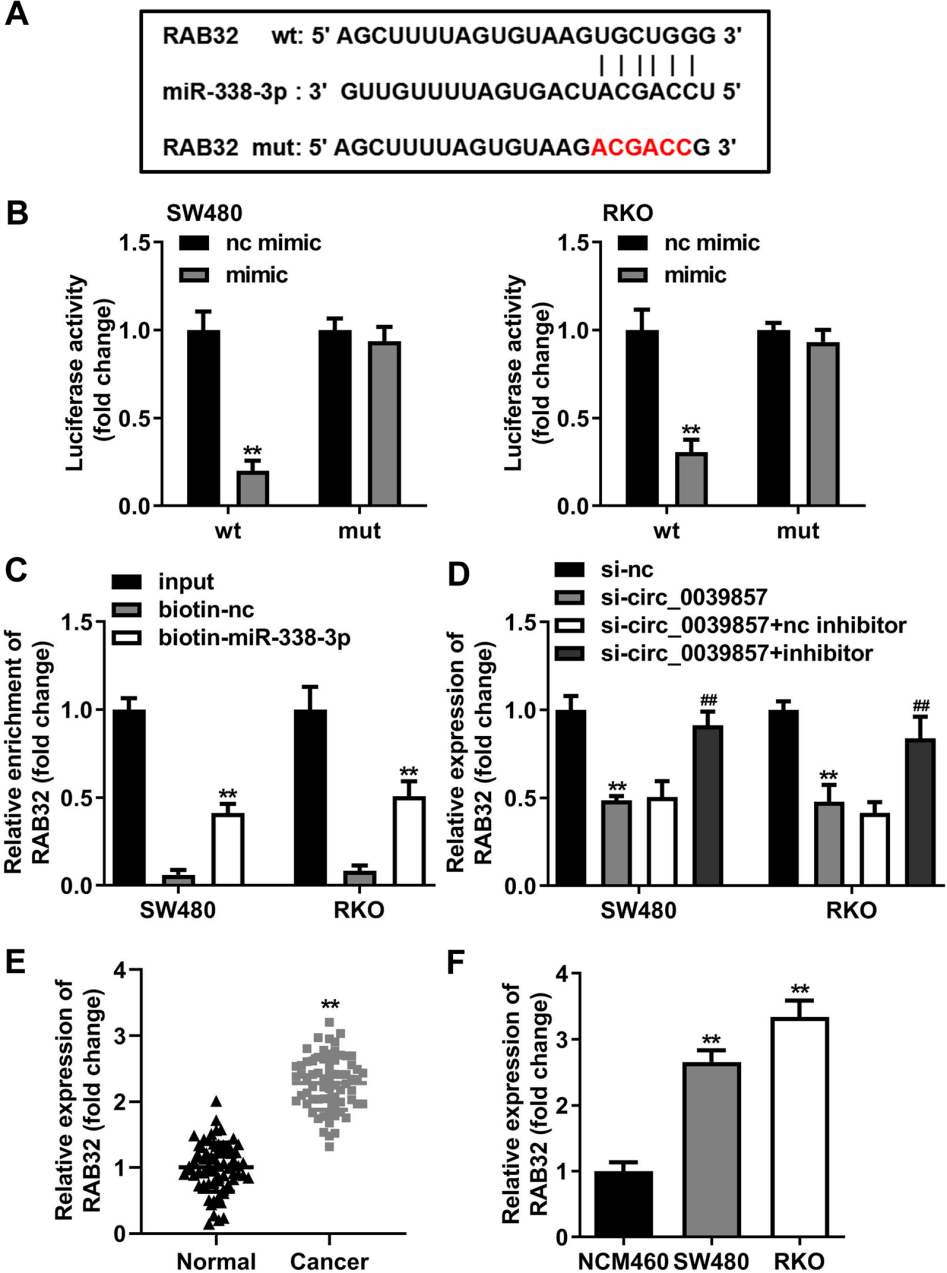
miR-338-3p targets and negatively regulates the expression of RAB32. **A** Complementary binding sequence between miR-338-3p and RAB32. **B** Dual-luciferase reporter gene assay. **C** RNA pull-down assay. **D** The regulatory effect of circ_0039857 on RAB32. **E** The expression level of RAB32 in tissues. **F** The expression level of RAB32 in cells. **P < 0.01

With the aim of exploring miR-338-3p mechanism in CRC, we predicted the downstream target genes of the miRNA. Bioinformatics analysis suggested that miR-338-3p had a complementary binding sequence ofRAB32 (Fig. 7A). The luciferase activity of the cells co-transfected with RAB32-wt reporter vector and miR-338-3p mimic was dramatically inhibited in accordance with the dual-luciferase reporter assay. (Fig. 7B). Biotin-labeled miR-338-3p also obviously enriched the RAB32 by RNA pull-down analysis (Fig. 7C). These results explained that miR-338-3p targeted RAB32. Next, we verified whether circ_0039857 regulated RAB32 through miR-338-3p. Knockdown of circ_0039857 conspicuously inhibited the expression of RAB32 in SW480 and RKO cells. However, when miR-338-3p expression was inhibited, the expression of RAB32 was restored (Fig. 7D). These captioned that circ_0039857 regulated RAB32 via targeting miR-338-3p. Finally, the expression of RAB32 in CRC was measured. A significant increase of RAB32 was observed in CRC tissues (Fig. 7E) and cells (Fig. 7F). These results revealed that RAB32 was a direct target of miR-338-3p.

### miR-338-3p regulates CRC cell proliferation and apoptosis via RAB32

**Fig. 8:**
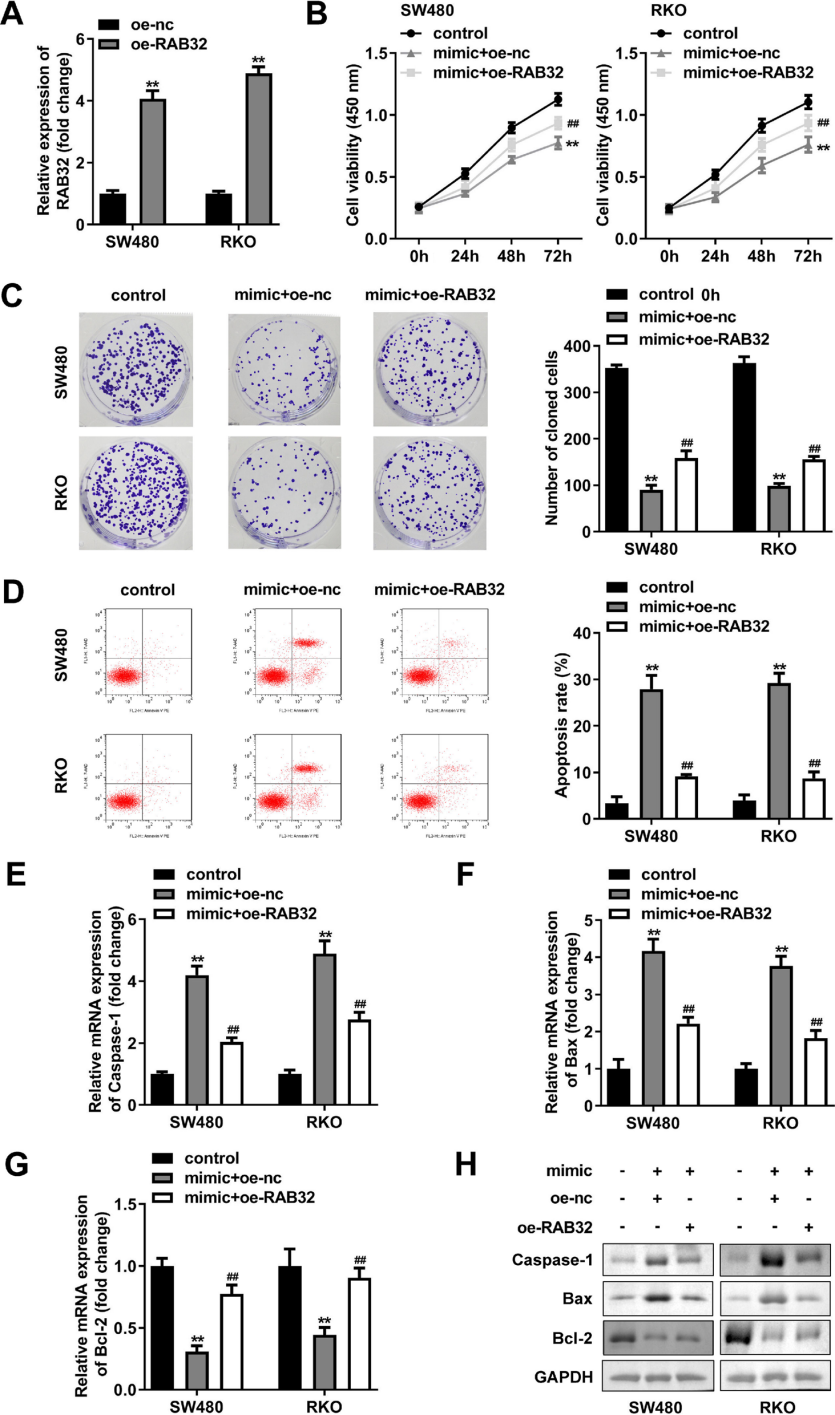
miR-338-3p regulates CRC cell proliferation and apoptosis via RAB32. **A** The transfection efficiencies of RAB32 overexpression plasmid vector in SW480 and RKO. **B** CCK-8 assay was performed to measure the proliferation ability of the cells. **C** The clonogenic ability of the cells. **D** The apoptosis rate of the cells was analyzed by flow cytometry. **E** The mRNA expression of caspase-1. **F** The mRNA expression of Bax. **G** The mRNA expression of Bcl-2. **H** The protein expression of caspase-1, Bax and Bcl-2. The membrane was cropped according to the molecular weight of the protein before the incubation with primary antibodies. GAPDH was used as a control. Full-length blots/gels are presented in Additional file 3: Fig. S3. **P < 0.01. ##*P* < 0.01

Finally, cells were transfected with a RAB32 overexpression plasmid vector to examine the mechanism of miR-338-3p/RAB32 axis in CRC. Based on RT-qPCR results, RAB32 was obviously up-regulated in the cells transfected with oe-RAB32 vectors, indicating that the cells were successfully transfected (Fig. 8A). Subsequently, RAB32 overexpression plasmid and miR-338-3p mimic were applied to co-transfect SW480 and RKO cells for rescue experiments, and NC was used as a control. RAB32 overexpression significantly suppressed the anti-proliferative ability of miR-338-3p mimic by CCK-8 and clone formation experiments. (Figs. 8B and C). In addition, RAB32 overexpression also suppressed the pro-apoptotic ability of miR-338-3p overexpression (Fig. 8D–H). To sum up, miR-338-3p affected CRC cell behavior through RAB32.

## Discussion

CRC is a frequent type of cancer nowadays. At present, the clinical treatment of CRC adopts a comprehensive treatment mode mainly based on surgery. In the past few decades, despite great progress in surgical techniques, chemotherapy and radiotherapy etc., its prognosis is still not optimistic [25, 26]. According to statistics, approximately 880,000 people died of CRC in 2018. CRC occurrence and development are caused by complex biological processes, including gene mutation, imbalance of cell proliferation, differentiation and apoptosis. [27]. Therefore, further searching for high-sensitivity, high-specificity, and high-stability diagnostic markers and possible targets for clinical treatment is particularly important for the development of CRC.

CircRNA is closed into a loop by covalent bonds between bases and not easy to be degraded by exonuclease (RNase R), so it remains stable in vivo[28]. Based on current research reports, the role of circRNAs in the tumor field has become a research hotspot. CircRNAs are abnormally expressed in tumor cell lines, suggesting that circRNAs are critical for tumorigenesis and progression [29]. Dysregulation of circRNAs has been identified as biomarkers of cancer progression in CRC. For example, circ_0006174 promotes CRC progression by targeting miR-138-5p/MACC1 [30]. Colon cancer cell progression and cisplatin resistance can be accelerated by circ_0020095/miR-487a-3p/SOX9 axis [31]. circTBL1XR1 is highly expressed in CRC and regulates Smad7 by sponging miR-424, which contributes to CRC malignancy [32]. It appears that circRNAs have indispensable roles in the development of CRC. However, the circRNAs network has not been fully elucidated and still needs further studies.

Based on our study, we found that circ_0039857 was greatly increased in CRC. Furthermore, ROC curve analysis showed that circ_0039857is of great value for the diagnosis of CRC. Apart from that, we also verified the stability of circ_0039857 in cells. Compared with linear RNA, circ_0039857 has higher stability. In CRC cells, loss-of-function experiments were conducted to investigate the biological function of circ_0039857. We observed that circ_0039857 knockdown obviously inhibited the cell proliferation and promoted apoptosis in CRC. Collectively, these data explained that circ_0039857 had an important biological function in CRC and acted as a tumor promoter. Nevertheless, the specific mechanism of circ_0039857 remains to be clarified.

With the in-depth understanding of circRNAs, scholars have found that circRNAs can reduce the inhibitory effect of microRNAs on their target genes, which is achieved through the competitive binding of miRNAs by numerous MERs [33]. At present, the most popular mechanism of circRNAs is that they function as sponges for miRNAs, releasing or alleviating miRNAs inhibition on their downstream target genes, thereby regulating important signaling pathways in the development of tumors [34]. The main function of miRNA is to inhibit the translation of target mRNA at the post-transcriptional level, thereby extensively participating in various biological processes [35]. Therefore, exploring the regulatory network of circRNA–miRNA–mRNA may contribute to understanding CRC progression and provide promising targets for the treatment of CRC.

In this study, we found that circ_0039857 targeted miR-338-3p-RAB32 axis. In addition, circ_0039857 positively regulated the RAB32 by targeting miR-338-3p. Rescue experiments suggested that miR-338-3p reversed the function of circ_0039857 as well as RAB32 on CRC cell behavior. Our data supported that circ_0039857 sponged miR-338-3p to promote RAB32 expression, thereby regulating cell progression in CRC cells.

To sum up, as a tumor-promoting factor, circ_0039857 was significantly upregulated in CRC. Circ_0039857 knockdown suppressed the malignant biological behavior by targeting the miR-338-3p/RAB32 axis in CRC cells. These results may help us to better understand the circRNA–miRNA–mRNA network in CRC progression, which may provide potential biomarkers and targets for future CRC therapy.

## Supplementary Information

**Additional file 1**. Full-length blots/gels

**Additional file 2**. Full-length blots/gels.

**Additional file 3**. Full-length blots/gels.

## Data Availability

All data generated or analyzed during this study are included in this published article.

## Abbreviations

CRC: Colorectal cancer
circRNAs: Circular RNAs
miRNAs: MicroRNAs
