# Supplementary material for: The hsa_circ_0039857/miR-338-3p/RAB32 axis promotes the malignant progression of colorectal cancer
Source: BMC Gastroenterol. 2022 Dec 20;22:530. doi: 10.1186/s12876-022-02622-1 (PMC9764720; doi:10.1186/s12876-022-02622-1)
Supplement: Supplementary file 2 — Additional file 2. Full-length blots/gels. [file 12876_2022_2622_MOESM2_ESM.pdf]

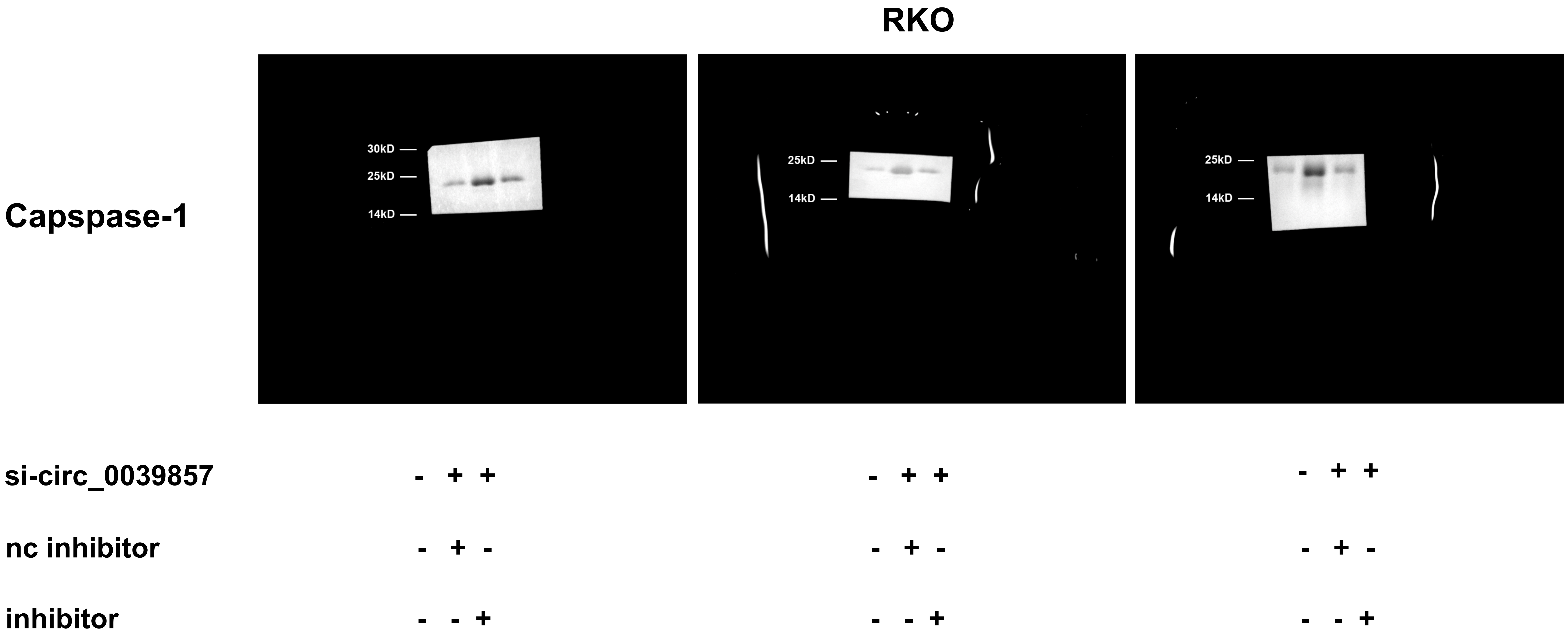
RKO
Capspase-1
30kD
25kD
14kD
25kD
14kD
25kD
14kD
si-circ_0039857
- + +
- + +
- + +
nc inhibitor
- + -
- + -
- + -
inhibitor
- - +
- - +
- - +

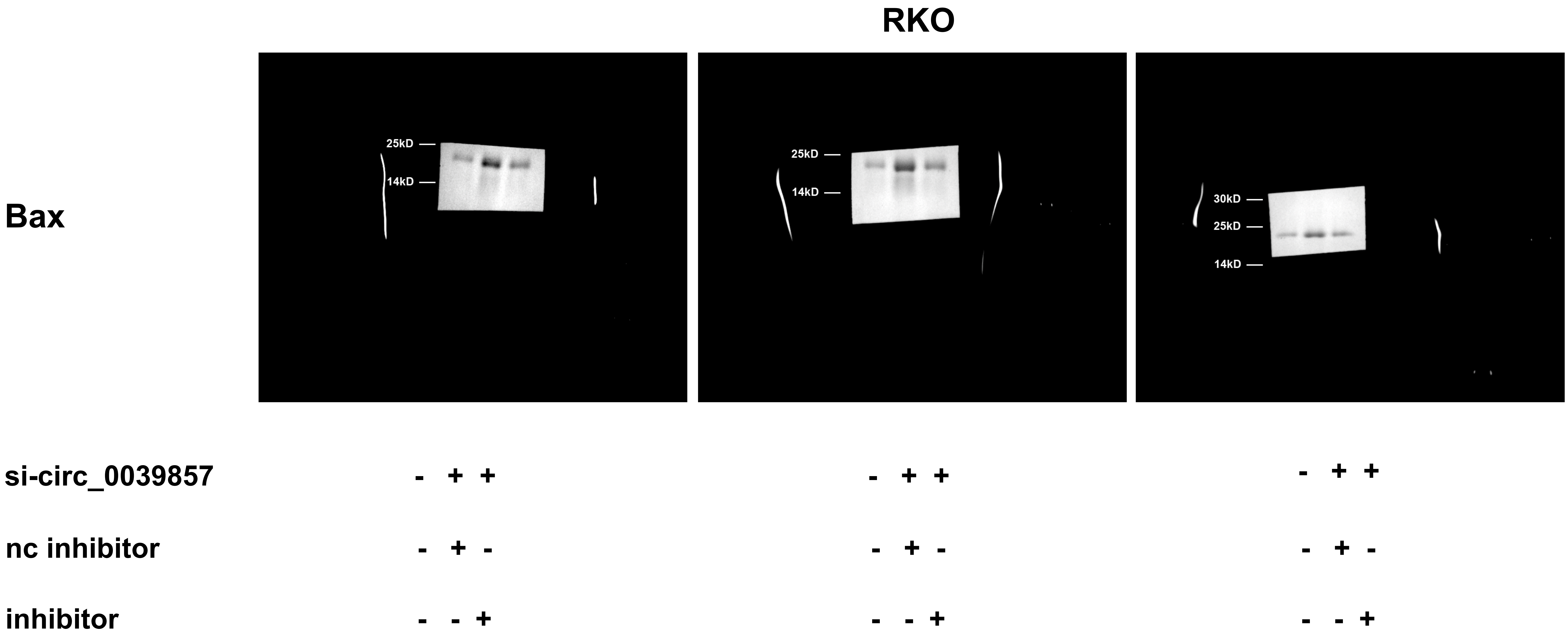
RKO
Bax
25kD
14kD
25kD
14kD
30kD
25kD
14kD
si-circ_0039857 - + + - + + - + +
nc inhibitor - + - - + - - + -
inhibitor - - + - - + - - +

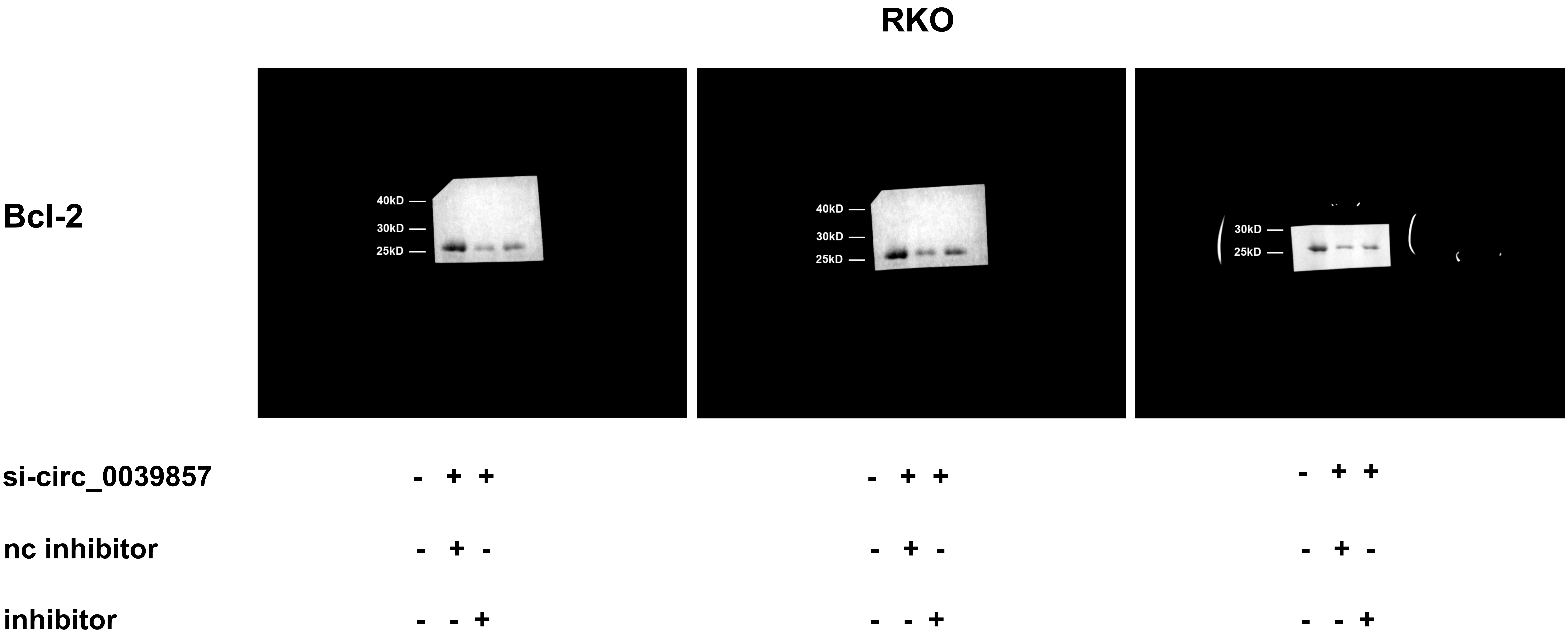
RKO
Bcl-2
40kD
30kD
25kD
si-circ_0039857 - + + - + + - + +
nc inhibitor - + - - + - - + -
inhibitor - - + - - + - - +

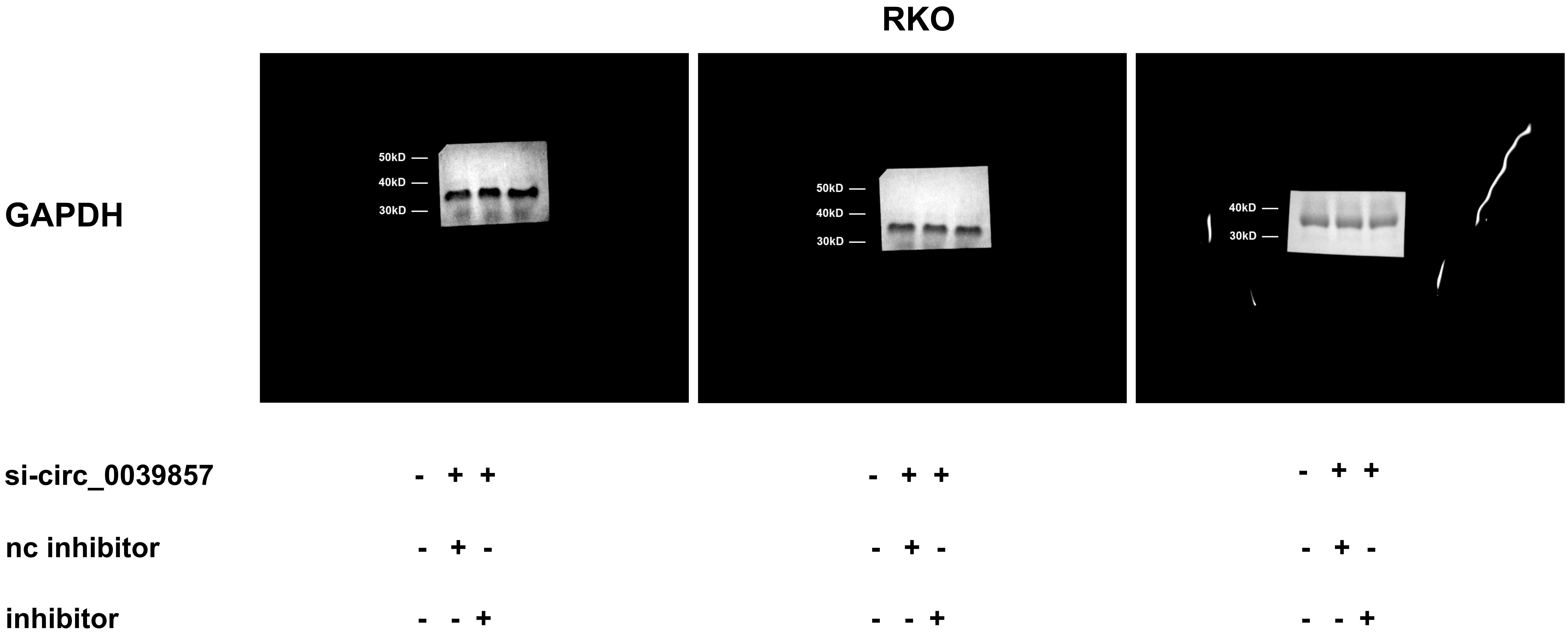
RKO
GAPDH
50kD
40kD
30kD
50kD
40kD
30kD
40kD
30kD
si-circ_0039857
- + +
- + +
- + +
nc inhibitor
- + -
- + -
- + -
inhibitor
- - +
- - +
- - +

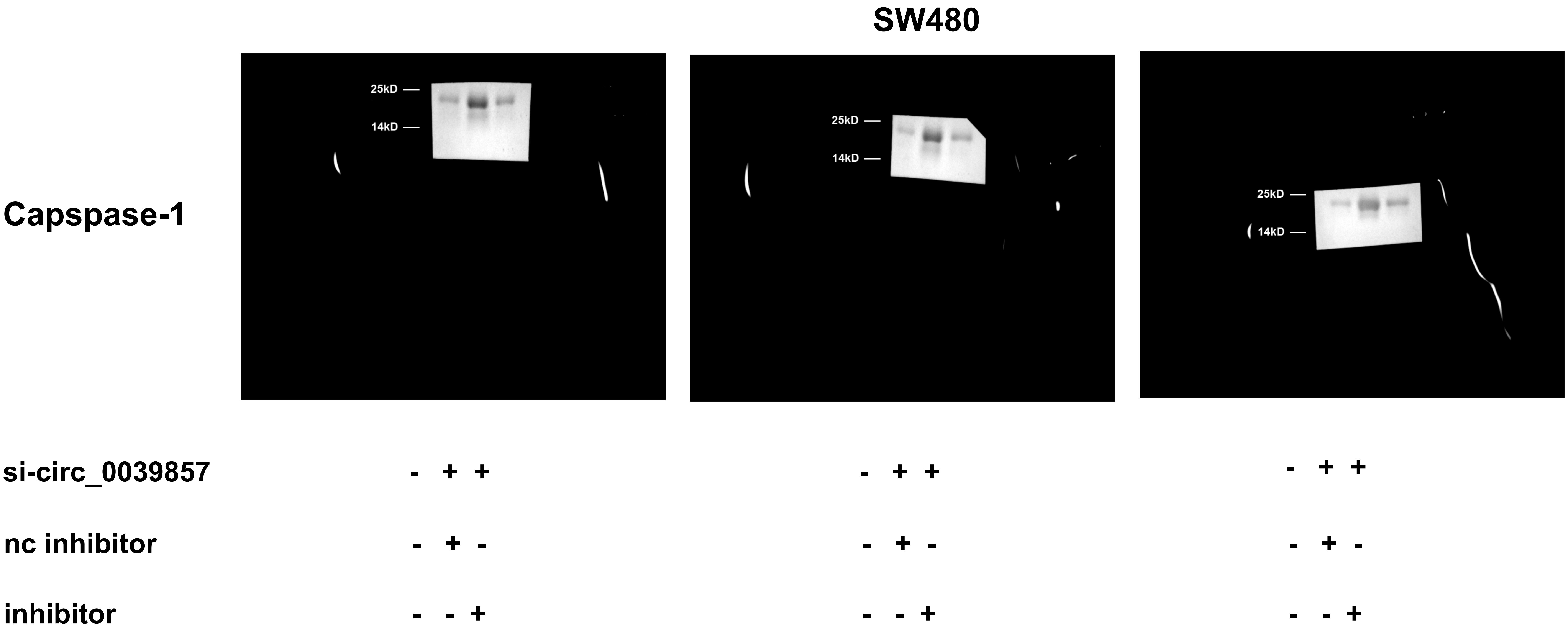
SW480
Capspase-1
25kD
14kD
25kD
14kD
25kD
14kD
si-circ_0039857 - + + - + + - + +
nc inhibitor - + - - + - - + -
inhibitor - - + - - + - - +

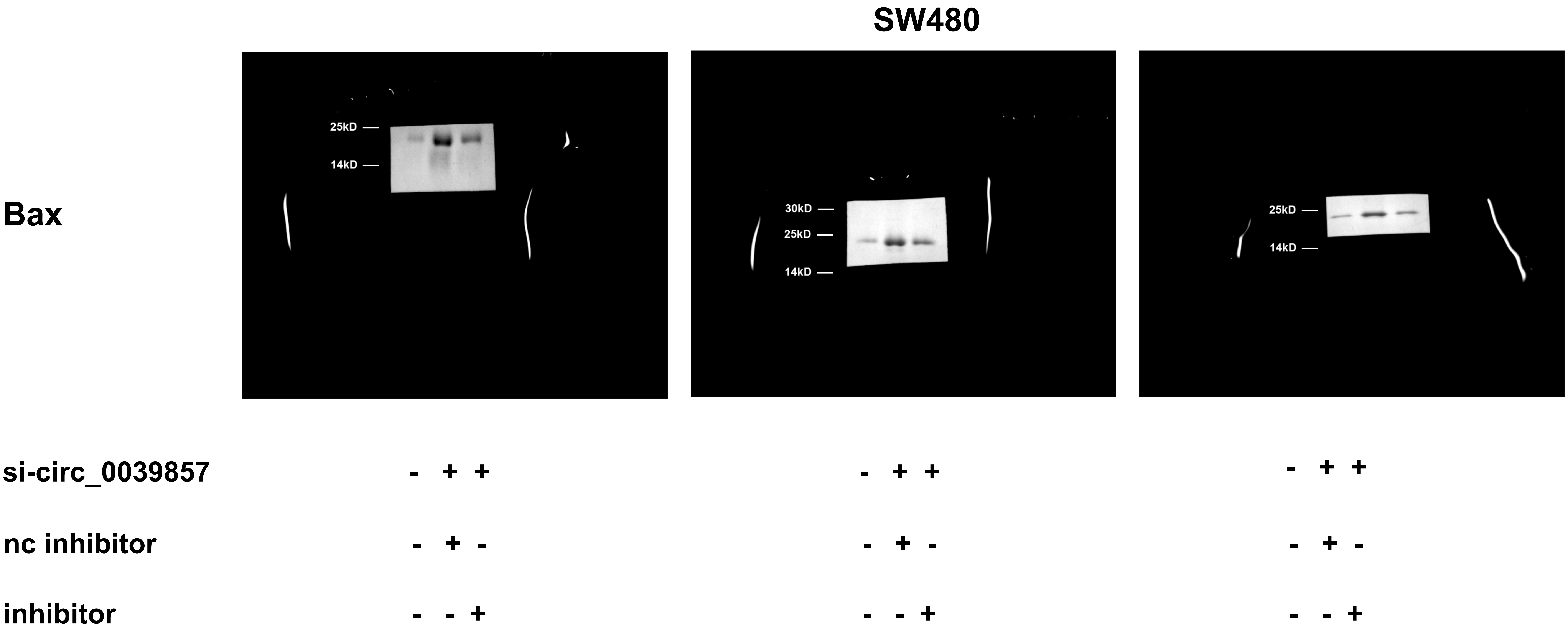
SW480
Bax
25kD
14kD
30kD
25kD
14kD
25kD
14kD
si-circ_0039857
- + +
- + +
- + +
nc inhibitor
- + -
- + -
- + -
inhibitor
- - +
- - +
- - +

SW480

Bcl-2

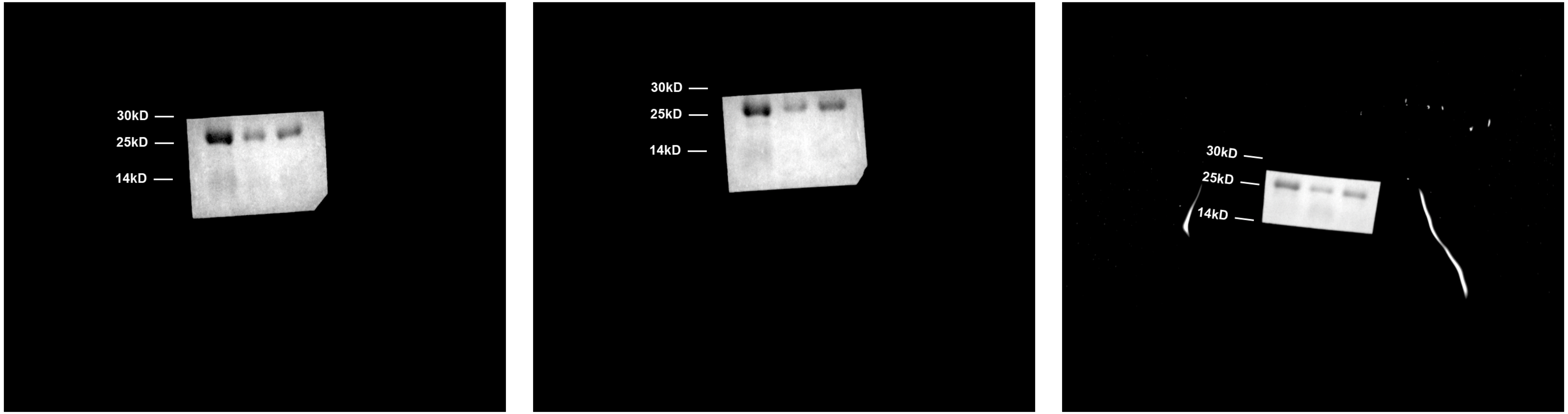

si-circ_0039857	-	+	+	-	+	+	-	+	+
nc inhibitor	-	+	-	-	+	-	-	+	-
inhibitor	-	-	+	-	-	+	-	-	+

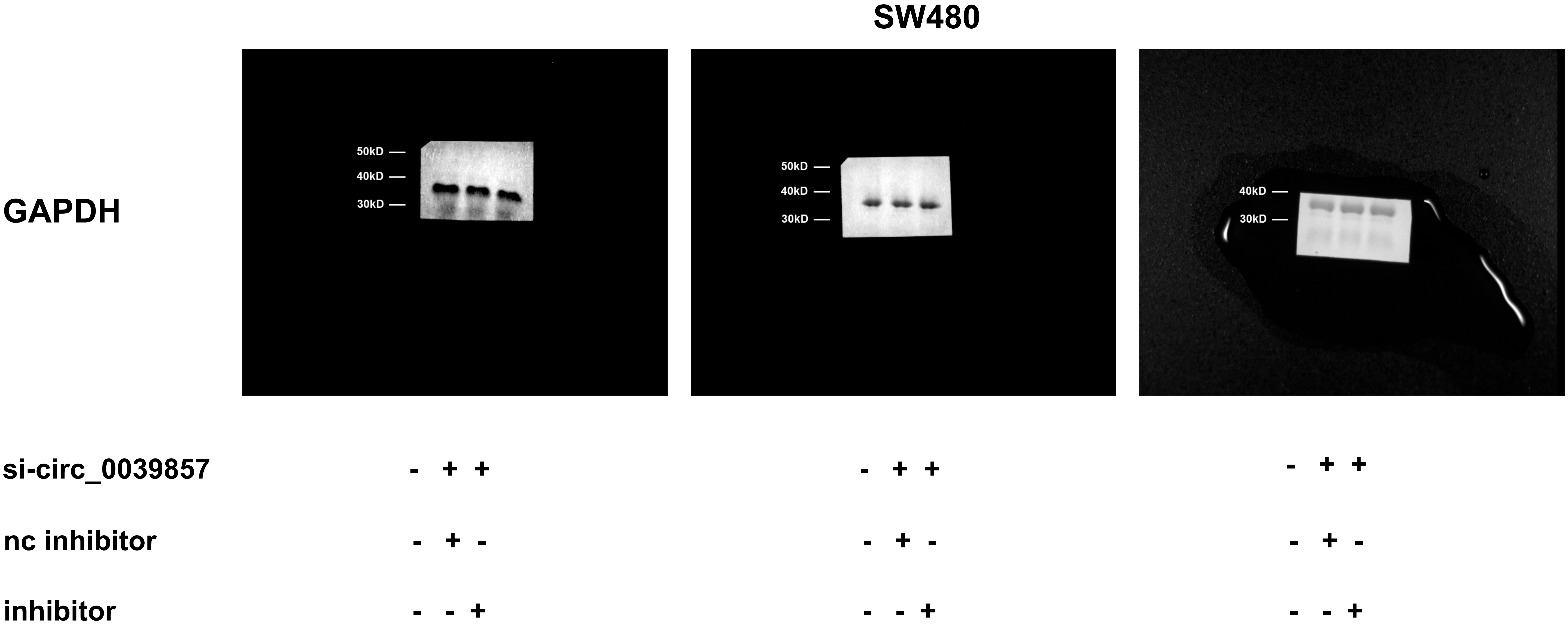
SW480
GAPDH
50kD
40kD
30kD
50kD
40kD
30kD
40kD
30kD
si-circ_0039857
- + +
- + +
- + +
nc inhibitor
- + -
- + -
- + -
inhibitor
- - +
- - +
- - +
