# Supplementary material for: The hsa_circ_0039857/miR-338-3p/RAB32 axis promotes the malignant progression of colorectal cancer
Source: BMC Gastroenterol. 2022 Dec 20;22:530. doi: 10.1186/s12876-022-02622-1 (PMC9764720; doi:10.1186/s12876-022-02622-1)
Supplement: Supplementary file 3 — Additional file 3. Full-length blots/gels. [file 12876_2022_2622_MOESM3_ESM.pdf]

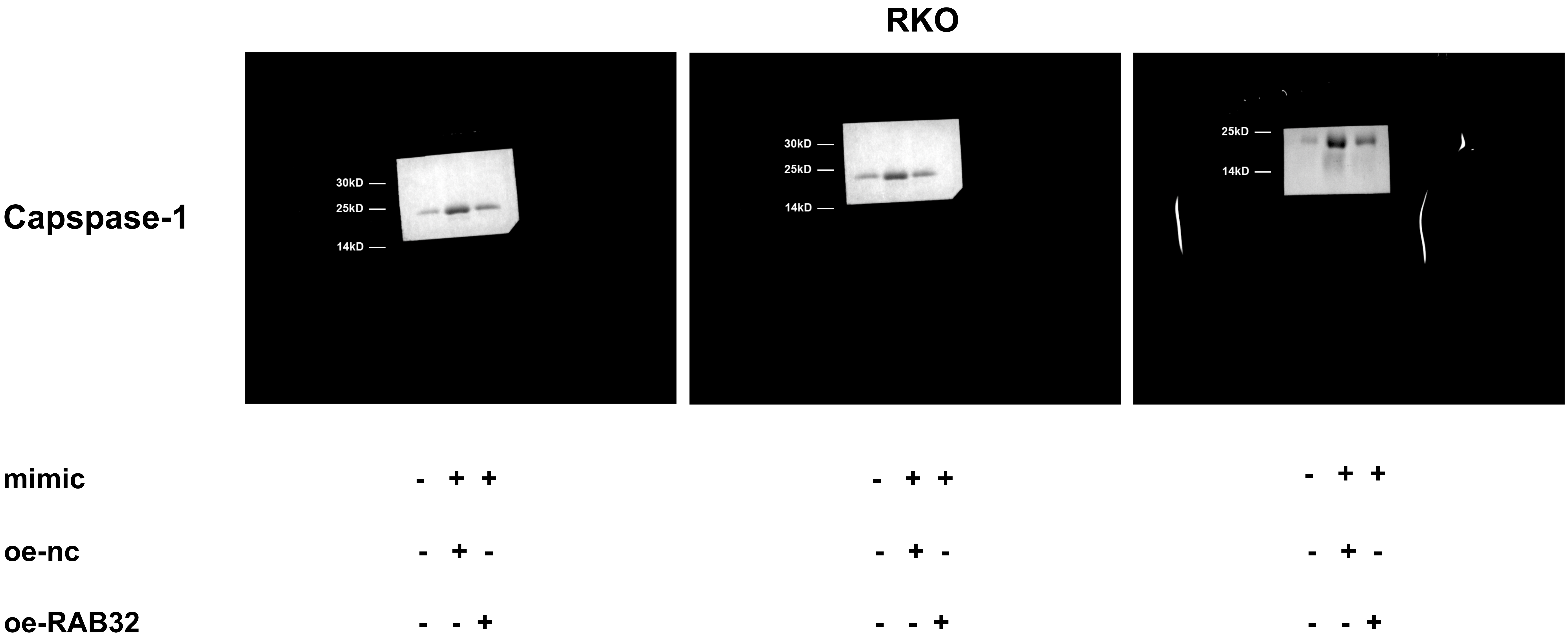
RKO
Capspase-1
30kD
25kD
14kD
mimic - + +
oe-nc - + -
oe-RAB32 - - +

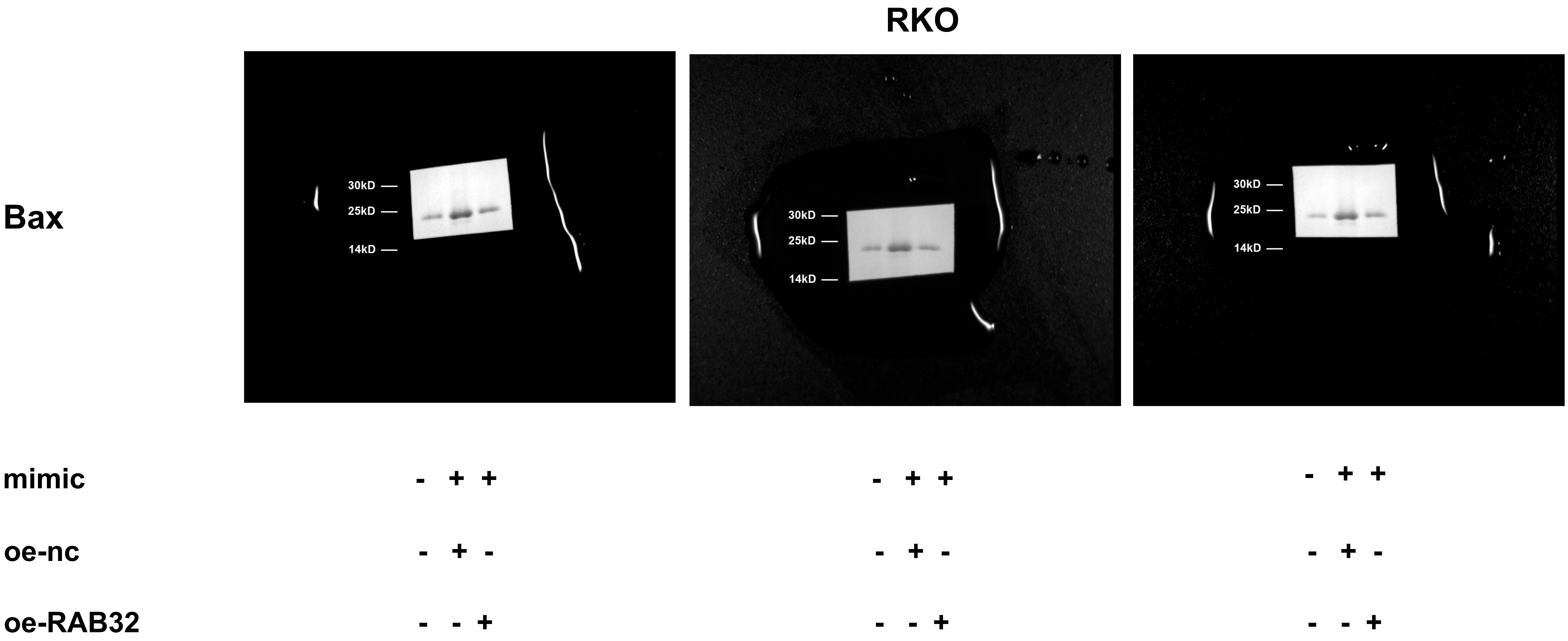
RKO
Bax
30kD
25kD
14kD
mimic - + +
oe-nc - + -
oe-RAB32 - - +

RKO

Bcl-2

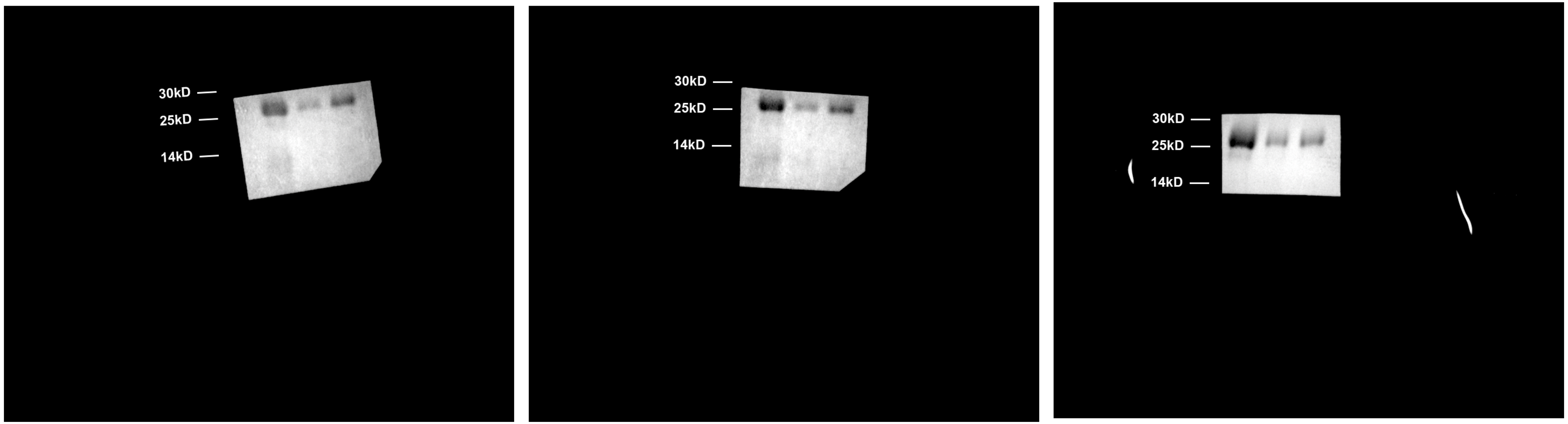

mimic	-	+	+	-	+	+	-	+	+
oe-nc	-	+	-	-	+	-	-	+	-
oe-RAB32	-	-	+	-	-	+	-	-	+

RKO

GAPDH

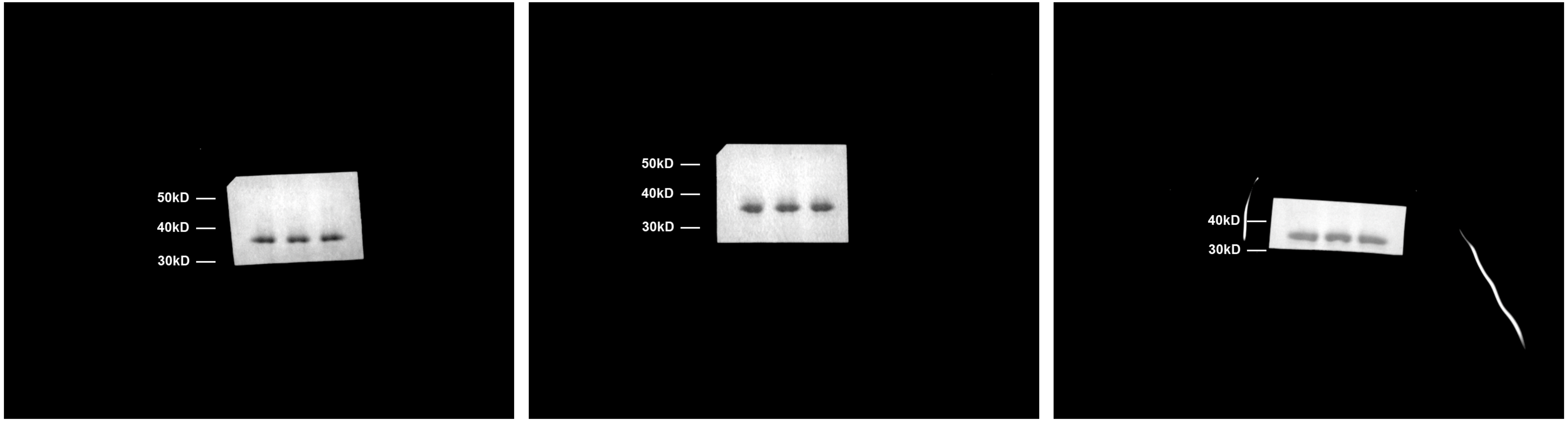

mimic	-	+	+	-	+	+	-	+	+
oe-nc	-	+	-	-	+	-	-	+	-
oe-RAB32	-	-	+	-	-	+	-	-	+

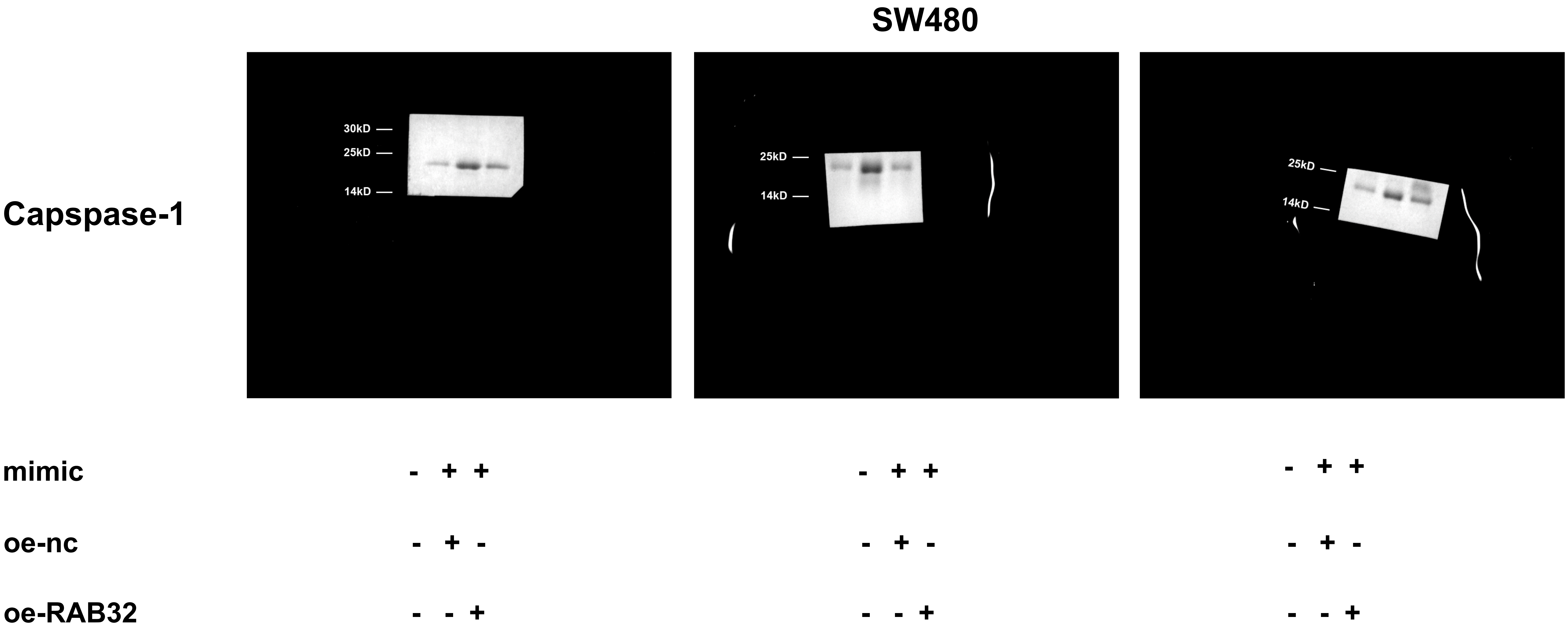
SW480
Capspase-1
30kD
25kD
14kD
25kD
14kD
25kD
14kD
mimic - + + - + + - + +
oe-nc - + - - + - - + -
oe-RAB32 - - + - - + - - +

SW480

Bax

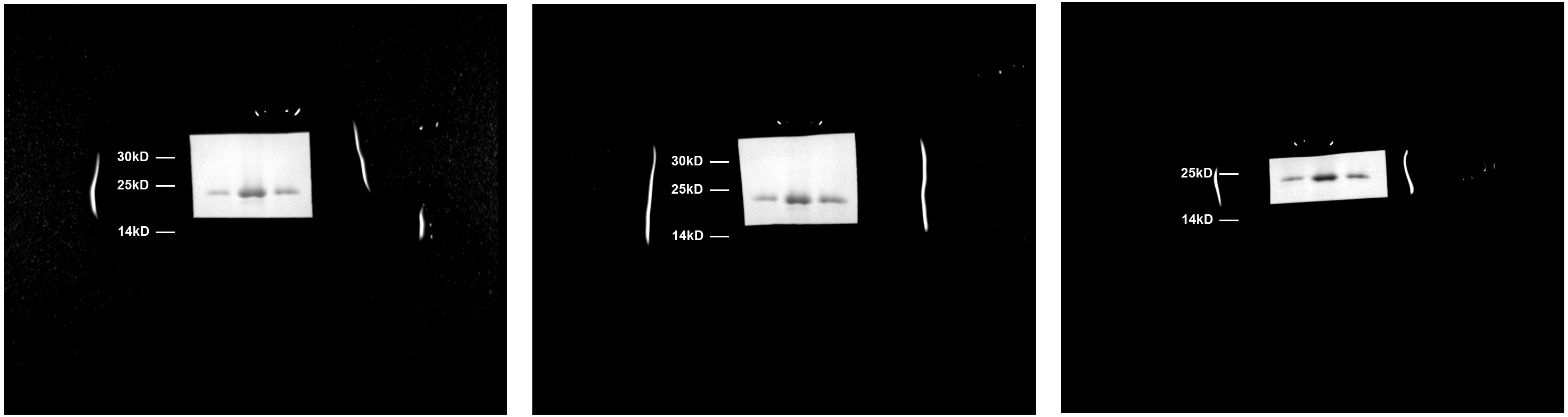

mimic	- + +	- + +	- + +
oe-nc	- + -	- + -	- + -
oe-RAB32	- - +	- - +	- - +

SW480

Bcl-2

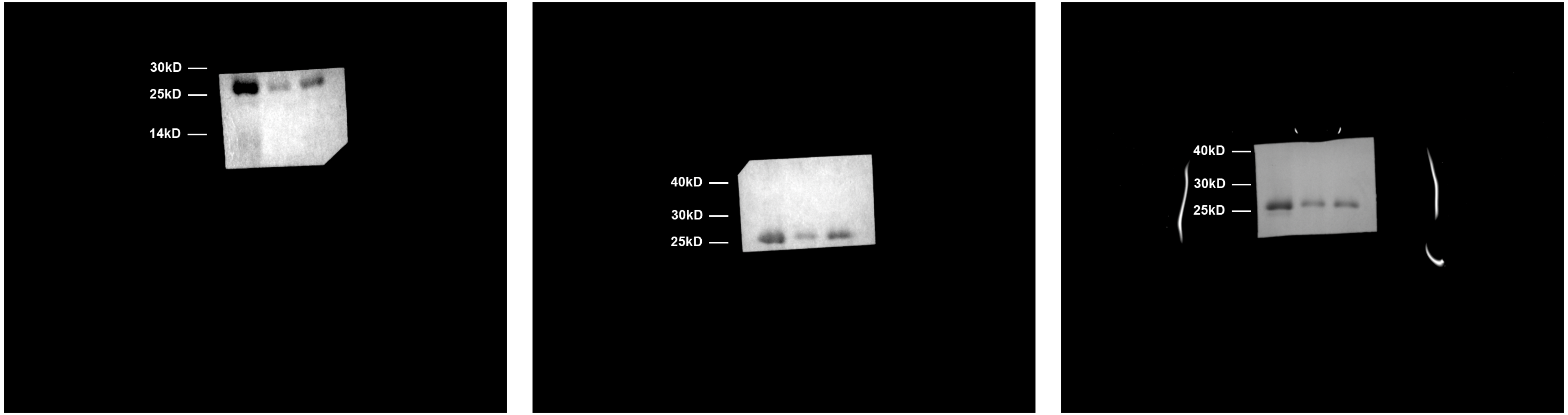

**mimic**	-	+	+	-	+	+	-	+	+
**oe-nc**	-	+	-	-	+	-	-	+	-
**oe-RAB32**	-	-	+	-	-	+	-	-	+

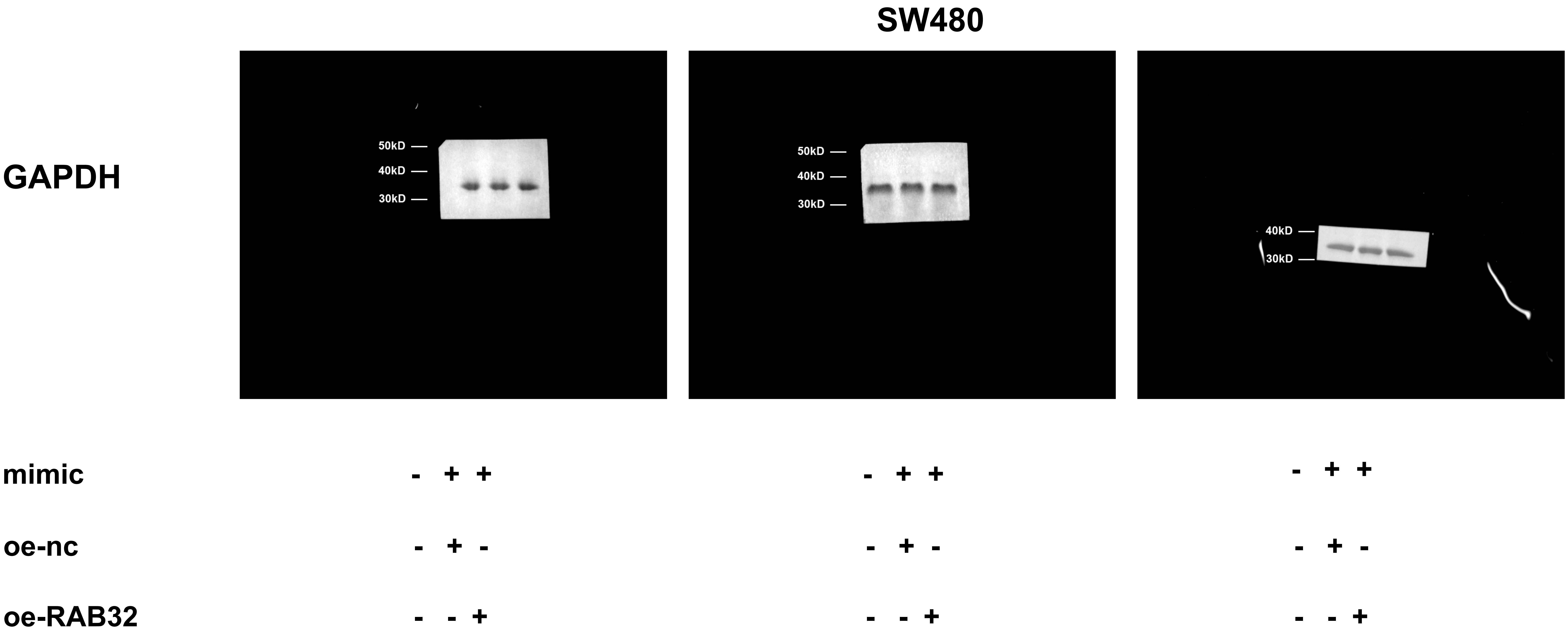
SW480
GAPDH
50kD
40kD
30kD
mimic
- + +
oe-nc
- + -
oe-RAB32
- - +
